# S100A6 inhibits MDM2 to suppress breast cancer growth and enhance sensitivity to chemotherapy

**DOI:** 10.1186/s13058-023-01657-w

**Published:** 2023-05-22

**Authors:** Mengxin Qi, Xianglan Yi, Baohui Yue, Mingxiang Huang, Sheng Zhou, Jing Xiong

**Affiliations:** Institute of Pathology, Tongji Hospital, Tongji Medical College, Huazhong University of Science and Technology, Wuhan, 430030 China

**Keywords:** Breast cancer, Chemosensitivity, S100A6, MDM2, HAUSP, Ubiquitination

## Abstract

**Background:**

S100A6 and murine double minute 2 (MDM2) are important cancer-related molecules. A previous study identified an interaction between S100A6 and MDM2 by size exclusion chromatography and surface plasmon resonance experiments. The present study investigated whether S100A6 could bind to MDM2 in vivo and further explored its functional implication.

**Methods:**

Co-immunoprecipitation, glutathione-*S*-transferase pull-down assay, and immunofluorescence were performed to determine the in vivo interaction between S100A6 and MDM2. Cycloheximide pulse-chase assay and ubiquitination assay were performed to clarify the mechanism by which S100A6 downregulated MDM2. In addition, clonogenic assay, WST-1 assay, and flow cytometry of apoptosis and the cell cycle were performed and a xenograft model was established to evaluate the effects of the S100A6/MDM2 interaction on growth and paclitaxel-induced chemosensitivity of breast cancer. The expressions of S100A6 and MDM2 in patients with invasive breast cancer were analyzed by immunohistochemistry. In addition, the correlation between the expression of S100A6 and the response to neoadjuvant chemotherapy was statistically analyzed.

**Results:**

S100A6 promoted the MDM2 translocation from nucleus to cytoplasm, in which the S100A6 bound to the binding site of the herpesvirus-associated ubiquitin-specific protease (HAUSP) in MDM2, disrupted the MDM2–HAUSP–DAXX interactions, and induced the MDM2 self-ubiquitination and degradation. Furthermore, the S100A6-mediated MDM2 degradation suppressed the growth of breast cancer and enhanced its sensitivity to paclitaxel both in vitro and in vivo. For patients with invasive breast cancer who received epirubicin and cyclophosphamide followed by docetaxel (EC-T), expressions of S100A6 and MDM2 were negatively correlated, and high expression of S100A6 suggested a higher rate of pathologic complete response (pCR). Univariate and multivariate analyses showed that the high expression of S100A6 was an independent predictor of pCR.

**Conclusion:**

These results reveal a novel function for S100A6 in downregulating MDM2, which directly enhances sensitivity to chemotherapy.

**Supplementary Information:**

The online version contains supplementary material available at 10.1186/s13058-023-01657-w.

## Background

As a multifunctional oncoprotein, murine double minute 2 (MDM2) is found to be overexpressed in patients with a variety of cancers [1], including breast cancer [2]. Overexpressed MDM2 is associated with disease progression and chemotherapy resistance, under influence of which the prognosis of patients with cancers is poor [3]. MDM2 plays a central role in regulating p53, which is a tumor suppressor. By binding to p53, MDM2 can inhibit the p53-mediated transcription and promote p53 ubiquitination and degradation [4]. In addition to p53, MDM2 interacts with multiple other molecules [5]. Over the past few years, studies on p53-independent pathways and activities of MDM2 have sharply increased [6, 7].

Expression of MDM2 can be regulated through multiple pathways, such as gene amplification, p53-mediated transcriptional induction, and post-translational regulation by self-ubiquitination [8]. MDM2 is an E3 ubiquitin ligase in the RING finger protein family and is its own substrate [9]. MDM2 self-ubiquitination has been a research hotspot for a long time [10]. The E3 ligase activity of MDM2 is in charge of MDM2 self-ubiquitination and is related to some cellular signaling pathways and molecular events. For instance, the MDM2 homolog MDM4 can bind to the RING domain of MDM2 and reduces its E3 ligase activity [11]. The herpesvirus-associated ubiquitin-specific protease (HAUSP) and death domain-associated protein (DAXX) interact with MDM2, forming a tertiary complex to weaken the MDM2 self-ubiquitination [12].

S100A6 (also known as Calcyclin) is a Ca^2+^-binding protein that belongs to the S100 protein family [13]. Similar to other members in the same family, S100A6 usually forms dimers with two EF-hand Ca^2+^-binding domains in each monomer [14]. Binding of Ca^2+^ will induce a conformational change in the S100A6 protein to expose the hydrophobic amino acid residues to interact with the target proteins [15]. There are several S100A6-target proteins, such as Lysozyme, Caldesmon, Tropomyosin, Annexin II, Annexin VI, Annexin XI, CacyBP/SIP, and Sgt1 [16–18]. These target proteins bind S100A6 to Ca^2+^ homeostasis, protein degradation control, and transcription factor regulation, thereby affecting cell proliferation, differentiation, apoptosis, stress response, and many other biological events [19].

MDM2 has been identified as another target protein of S100A6. Size exclusion chromatography and surface plasmon resonance experiments show that S100A6 binds directly to MDM2 in vitro [20]. However, the effect of S100A6 on MDM2 activity remains unclear. The present study examined whether S100A6 could bind to MDM2 in vivo and further explored the functional significance of this interaction. The interaction between S100A6 and MDM2 was demonstrated by co-immunoprecipitation, glutathione-*S*-transferase (GST) pull-down assay, and immunofluorescence. Further studies found that S100A6 bound to the binding site of the deubiquitinating enzyme HAUSP in MDM2, disrupted the MDM2–HAUSP–DAXX interactions, and induced MDM2 self-ubiquitination and degradation. Moreover, the S100A6-mediated MDM2 degradation suppressed the growth of breast cancer and enhanced sensitivity to paclitaxel both in vitro and in vivo. For patients with invasive breast cancer who received epirubicin and cyclophosphamide followed by docetaxel (EC-T), the expressions of S100A6 and MDM2 were negatively correlated, and S100A6 was a predictive biomarker for the efficacy of neoadjuvant chemotherapy. This study proposes a novel and important function of S100A6 in regulating MDM2 that directly affects the growth of tumor cells and their sensitivity to chemotherapy, which is of high value in clinical use.

## Methods

### Cell lines and reagents

In this study, six breast cancer cell lines (SUM159, MCF-7, ZR-75-1, MDA-MB-231, MDA-MB-468, and HCC1937) were selected and supplied by the American Type Culture Collection (ATCC) and then cultured as instructed. In addition, paclitaxel was purchased from Sigma-Aldrich, and the stock solution was diluted in dimethyl sulfoxide (DMSO) at 4 mM. Cycloheximide (CHX, 0.1 mM) and MG132 (10 mM) were purchased from MCE and were diluted in DMSO before use.

### Plasmids and transfection

Various truncated or mutated human S100A6, MDM2, and HAUSP constructs were generated by using polymerase chain reaction (PCR) and then cloned into pCMV-Flag, pCMV-Myc, or pCMV-HA expression vectors (Clontech). Meanwhile, various truncated human S100A6 and MDM2 constructs were generated by PCR and cloned into T7 promotor-GST or T7 promotor-HA expression vectors (Clontech). MDM2 mutations included MDM2 C461S (substitution of Cys by Ser), MDM2 C464A (substitution of Cys by Ala), and MDM2 C478S (substitution of Cys by Ser). The cells were transfected with S100A6 siRNA (CCUCUCUGAGUCAAAUCCATT) with Lipofectamine 3000 (Invitrogen). The overexpression rate and knockdown effect were detected by western blot.

### Western blot

The expressions of S100A6, MDM2, HAUSP, DAXX, Ubiquitin, HA, GST, Flag, and Myc proteins were measured by western blot. The employed antibodies were listed as follows. S100A6 antibody (ab 181975), HAUSP antibody (ab 264422), HA tag antibody (ab 9110), GST-tag antibody (ab 138491), and Flag tag antibody (ab 205606) were purchased from Abcam. MDM2 antibodies were purchased from Abcam (ab 259265), Cell Signaling Technology (D1V2Z), and ABclonal (A0345). Ubiquitin antibody was purchased from ABclonal (A19686). Myc tag antibodies were purchased from Abcam (ab 32972) and Santa Cruz (sc-40). DAXX antibody was purchased from Santa Cruz (sc-8043). The concentrations of all antibodies were applied strictly according to the manufacturers’ instructions.

### Co-immunoprecipitation

S100A6 and MDM2 plasmids were co-transfected into MCF-7 cells. Twenty-four hour after transfection, cells were lysed in ice-cold immunoprecipitation lysis/wash buffer (Servicebio, 100 μL/1 × ${10}^{6}$ cells) at 4 °C for 30 min and then centrifuged at 12,000 rpm for 10 min. An equal amount of cell lysates (100 μL) were immunoprecipitated with anti-S100A6, anti-MDM2, anti-HAUSP, or IgG antibody and A/G agarose (Bimake, 25 μL) overnight at around 4 °C for at least 12 h. The cell lysates were discarded, the magnetic beads were retained and rinsed, and the protein was eluted in 1 × sodium dodecyl sulfate (SDS) loading buffer. Bound proteins were eluted and detected by western blot using corresponding antibodies.

### GST pull-down assay

The plasmids GST, GST-S100A6 or its fragments, and HA-MDM2 or its fragments were generated as described previously. These plasmids were transformed into Escherichia coli strain BL21 (DE3). The single colonies were selected and cultured in ampicillin-resistant liquid LB medium, shocked at 37 °C until the optical density reached 0.5, and then shocked again at 20–23 °C overnight with the addition of IPTG (0.5 mmol/L). After the recombinant proteins were obtained, HA-MDM2 or its fragments (carried His-tag) and GST-S100A6 or its fragments (carried GST-tag) were purified with a purification kit (His-tag or GST-tag, Biolinkedin) separately according to the instructions.

Approximately 100 µg of mixture composed of GST or GST-S100A6 or its fragments and HA-MDM2 or its fragments were immobilized in 100 µL of glutathione agarose (Biolinkedin) for incubation overnight at 4 °C under gentle rotation. The magnetic beads were retained, rinsed, and eluted in 1 × SDS loading buffer. Finally, the western blot was performed on the bound proteins.

### Immunofluorescence

MCF-7 cells were transfected with Myc-MDM2 and/or Flag-S100A6 plasmids for 24 h. They were rinsed with phosphate buffer saline (PBS), fixed with pre-chilled 4% paraformaldehyde for a quarter hour, and then permeabilized with 0.1% Triton X-100 for another quarter hour at ambient temperature. Next, they were blocked with 5% BSA for 60 min at the same temperature as above and then incubated with an anti-Myc rabbit antibody (1:100 dilution, Santa Cruz, sc-40) and anti-Flag mouse antibody (1:100 dilution, Abcam, ab 259,265) at 4 °C overnight. After that, they were rinsed with PBS (3 times × 5 min) and stained with Fluorescein (FITC)-conjugated AffiniPure Donkey Anti-Rabbit IgG (1:100 dilution, Jackson, 711-095-152) and Rhodamine Red-X-conjugated AffiniPure Donkey Anti-Mouse IgG (1:100 dilution, Jackson, 715-295-150) for about 60 min in the dark at room temperature. Again, they were rinsed, 3 times × 5 min, with PBS and then stained with diamamidine phenylindole (DAPI) for 15 min at 37 °C. Images were captured with a laser confocal microscope (EVIDENT, FV1000).

### CHX pulse-chase assay

Protein turnover of MDM2 was assessed by CHX pulse-chase assay. MCF-7 cells were co-transfected with MDM2 and S100A6/Flag-S100A6 or HA-HAUSP plasmids for 24 h. After that, the protein synthesis inhibitor CHX (MCE, 0.2 μM) was added 15, 30, 60, and 90 min before cell lysis. After CHX treatment, the cell lysates were prepared and the expression of MDM2 was detected by western blot.

### Ubiquitination assay in vivo

Cells were treated with the proteasome inhibitor MG132 (MCE, 0.5 μM) for 10 h before collection. Cell lysates, after low-temperature centrifugation, were then incubated with A/G magnetic beads and anti-MDM2 antibody on a rotator at low temperature overnight. The beads were washed, and the ubiquitinated products were eluted and detected by western blot using an anti-ubiquitin antibody.

### Ubiquitination assay in vitro

Full-length GST-S100A6 and HA-MDM2 and its fragments were expressed and purified as previously described in the GST pull-down assay. The in vitro ubiquitination assay was performed in a total reaction volume (approximately 100μL) consisting of 5 μg E1 (0.5 μg/μL), 5 μg UbcH5 (1 μg/μL), 50 μg HA-MDM2, 10 μg ubiquitin (1 μg/μL), 50 μg GST-S100A6, and 5 μg 10 × Buffer (1 μg/μL). E1, UbcH5, ubiquitin, and 10 × Buffer were purchased from Enzo Life Science. The in vitro reactions were carried out at 37 °C and 380 rpm for 60–90 min, followed by a western blot assay.

### Colony formation assay

S100A6, MDM2, or MDM2 and S100A6-transfected MCF-7 cells were prepared in a single-cell suspension. After that, 150 cells were seeded into a 6-well plate, which lasted for about 14 days so that colonies were generated. Paclitaxel was then added to the cell culture medium at various concentrations. After 48 h, the cells, with or without paclitaxel, were rinsed with PBS and then fixed with 4% paraformaldehyde for 30 min and stained with 0.5% crystal violet for about 20 min. After the dying solutions were removed carefully, the colonies generated previously were rinsed with PBS, counted, and calculated.

### WST-1 assay

The water-soluble tetrazolium (WST) salt assay was employed in this study to analyze the effect of S100A6 on paclitaxel-induced cytotoxicity. Cells transfected with S100A6, MDM2, or MDM2 and S100A6 were placed in 96-well plates for culture and then treated with paclitaxel at various concentrations for a day and a night (24 h) at 37 °C. The mixture of cells and WST-1 (25 μg/well) was incubated for another 4 h. At this time, the optical density was detected at 450 nm by using 620 nm as a reference.

### Flow cytometry of apoptosis and cell-cycle

Annexin-V staining followed by flow cytometry was employed to demonstrate the effects of paclitaxel and S100A6 on apoptosis. Cells with S100A6, MDM2, or MDM2 and S100A6 transfection and paclitaxel (100 μM for different times) treatment were harvested, rinsed twice with cold PBS, stained with FITC-Annexin-V and PI (BD Pharmingen) following the manufacture’s suggestions, and then detected by flow cytometry.

The cells were treated the same as above and then fixed in 70% ethanol for over 12 h. Then, they were centrifuged and rinsed with PBS twice. Finally, cells were dissolved in 200 μL of PI/RNase Staining Buffer (BD Pharmingen) and detected by flow cytometry to measure the DNA content.

### Xenograft model

Specific pathogen-free (SPF) nude mice (BALB/c, ~ 18 g, 4 weeks old) were randomly allocated to 8 groups. MCF-7 cells were infected with lentivirus expressing S100A6, MDM2, or MDM2 and S100A6. The stable transfected cells (2 × 10^6^ cells in 150 μL PBS) were subcutaneously inoculated into the right posterior flank of female SPF nude mice. Ten days later, the mice were treated with or without intraperitoneal injection of paclitaxel (20 mg/kg, 3 times/week, for 4 weeks). After treatment, the mice were humanely killed on day 38, and tumor tissues were used for western blot.

### Patients

A cohort of 107 patients with invasive breast cancer who received EC-T neoadjuvant chemotherapy (E: 100 mg/${\mathrm{m}}^{2}$ epirubicin administered intravenously i.v.), C: 800 mg/${\mathrm{m}}^{2}$ cyclophosphamide i.v., and T: 75 mg/${\mathrm{m}}^{2}$ docetaxel i.v.) during 2020–2022 at the Tongji Hospital of Huazhong University of Science and Technology were included in this study. The pathological diagnosis and other biological assessment of the biopsy before chemotherapy were collected and classified. Patients were categorized into three different molecular subtypes based on their gene expressions: Luminal (ER + and HER2 − ); HER2 + ; and TNBC (ER − , PR  − , and HER2 − ). Tumor response to chemotherapy was assessed after mastectomy and axillar y lymph-node excision or breast-conserving surgery. pCR refers to remitting the invasive tumor lesions by surgically removing the breast tissue and axillary lymph nodes after chemotherapy.

### Immunohistochemistry

Immunohistochemistry of S100A6 and MDM2 in tumor tissues was performed according to the previously published method mentioned in Reference [21]. Two independent pathologists who did not know the details of the patients evaluated the staining results. The expressions of S100A6 (1:2000 dilution, Abcam, ab 181,975) and MDM2 (1:1000 dilution, ABclonal, A0345) were scored using a modified histochemical score (H-score). The intensity of staining was graded as 0–3 (0: no staining, 1: weak staining, 2: moderate staining, 3: strong staining). The percentage of stained cells was expressed as 0–100%. Meanwhile, the H-score (0–300) was calculated by multiplying the intensity of staining and the percentage of stained cells. S100A6 or MDM2 was divided into two groups according to the H-score: a low expression (H-score < 100) and a high expression (H-score ≥ 100), with a cutoff score of 100.

### Statistical analysis

All data were determined after three or more independent experiments and expressed as the mean ± standard deviation (SD). They were processed and analyzed with ImageJ, GraphPad Prism, and SPSS, respectively. The normality of data distributions was subjected to the Shapiro–Wilk test. The independent sample T test was employed for comparison between two groups, while one-way or multi-factor analysis of variance was selected for comparisons of three or more groups. Immunofluorescence was analyzed with the Pearson’s correlation coefficient (PCC). Data from clinical samples were analyzed with the Chi-square test and Fisher’s exact test. *P < 0.05, **P < 0.01, ***P < 0.001.

## Results

### MDM2 is negatively regulated by S100A6

**Fig. 1:**
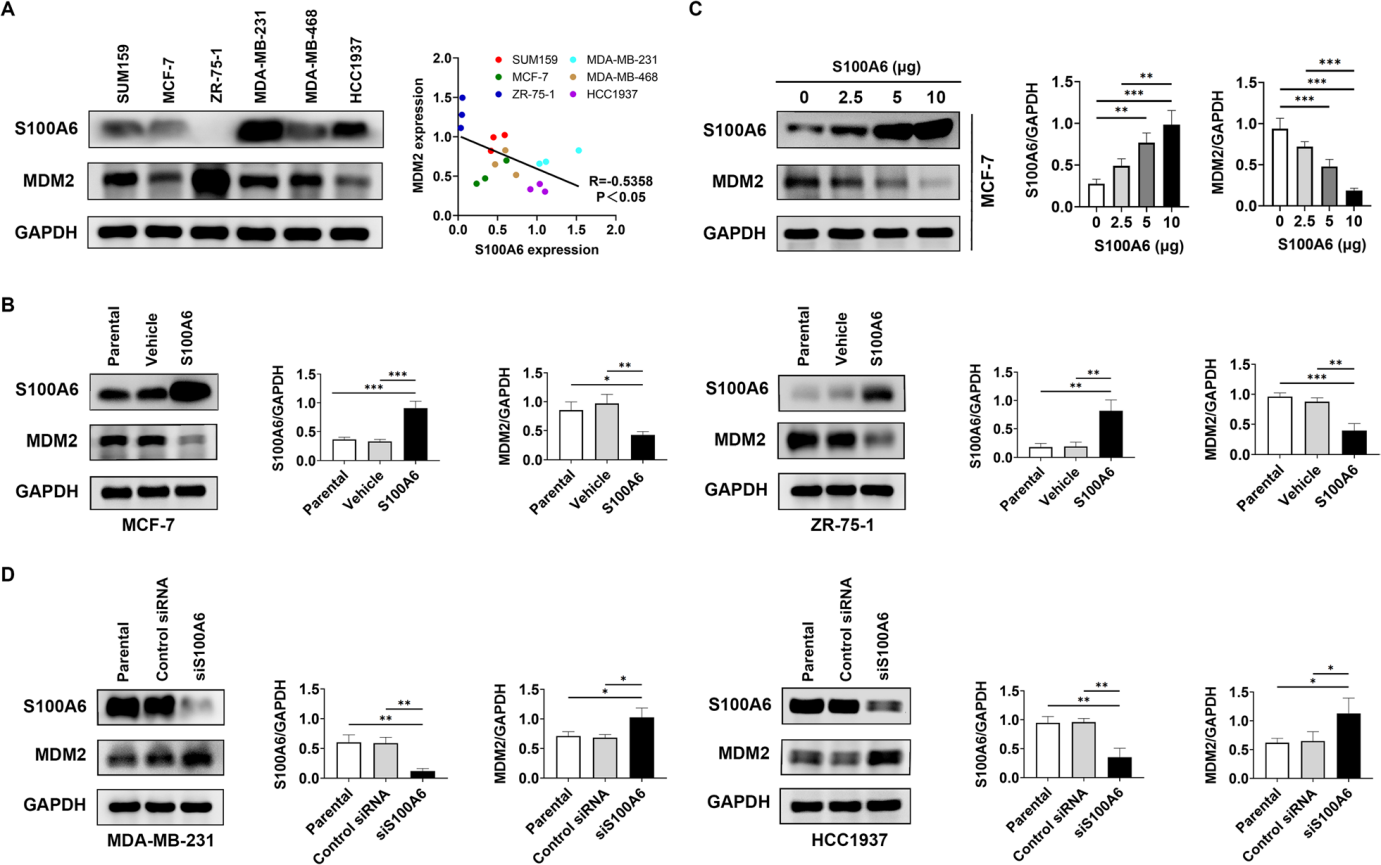
S100A6 inhibits MDM2 in breast cancer cell lines. **A** The expression levels of S100A6 and MDM2 in SUM159, MCF-7, ZR-75–1, MDA-MB-231, MDA-MB-468, and HCC1937. Correlation of the expressions of S100A6 and MDM2. **B** and **D** Expressions of MDM2 in MCF-7 and ZR-75–1 versus MDA-MB-231 and HCC1937 under the transfection of S100A6 (5 μg, S100A6 plasmid) and S100A6 siRNA (5 μg, siS100A6), respectively. **C** Dose–response expression of MDM2 in MCF-7 after S100A6 transfection. Histograms showed the densitometric analyses of indicated proteins. Data were shown as mean ± SD; *n* = 3 independent experiments. *P < 0.05, **P < 0.01, and ***P < 0.001

To determine whether the S100A6 was correlated with MDM2, six human breast cancer cell lines (SUM159, MCF-7, ZR-75-1, MDA-MB-231, MDA-MB-468, and HCC1937) were tested firstly to measure the expressions of S100A6 and MDM2. Western blot assay showed that a high expression of S100A6 was closely associated with a low expression of MDM2. MDA-MB-231 and HCC1937 expressed a high expression of S100A6 and low expression of MDM2; SUM159, MCF-7, ZR-75-1, and MDA-MB-468 showed a low expression of S100A6 and relatively high expression of MDM2 (Fig. 1A).

S100A6 transfection in MCF-7 and ZR-75-1 cells was performed to further confirm this correlation. S100A6 transfection downregulated MDM2 (Fig. 1B) in a dose-dependent manner (Fig. 1C). In addition, S100A6 was silenced using siRNA in MDA-MB-231 and HCC1937 cells. MDM2 expression was significantly upregulated in these S100A6-silenced cells (Fig. 1D). These results suggest that MDM2 is negatively regulated by S100A6.

### Binding between S100A6 and MDM2 both in vivo and in vitro

**Fig. 2:**
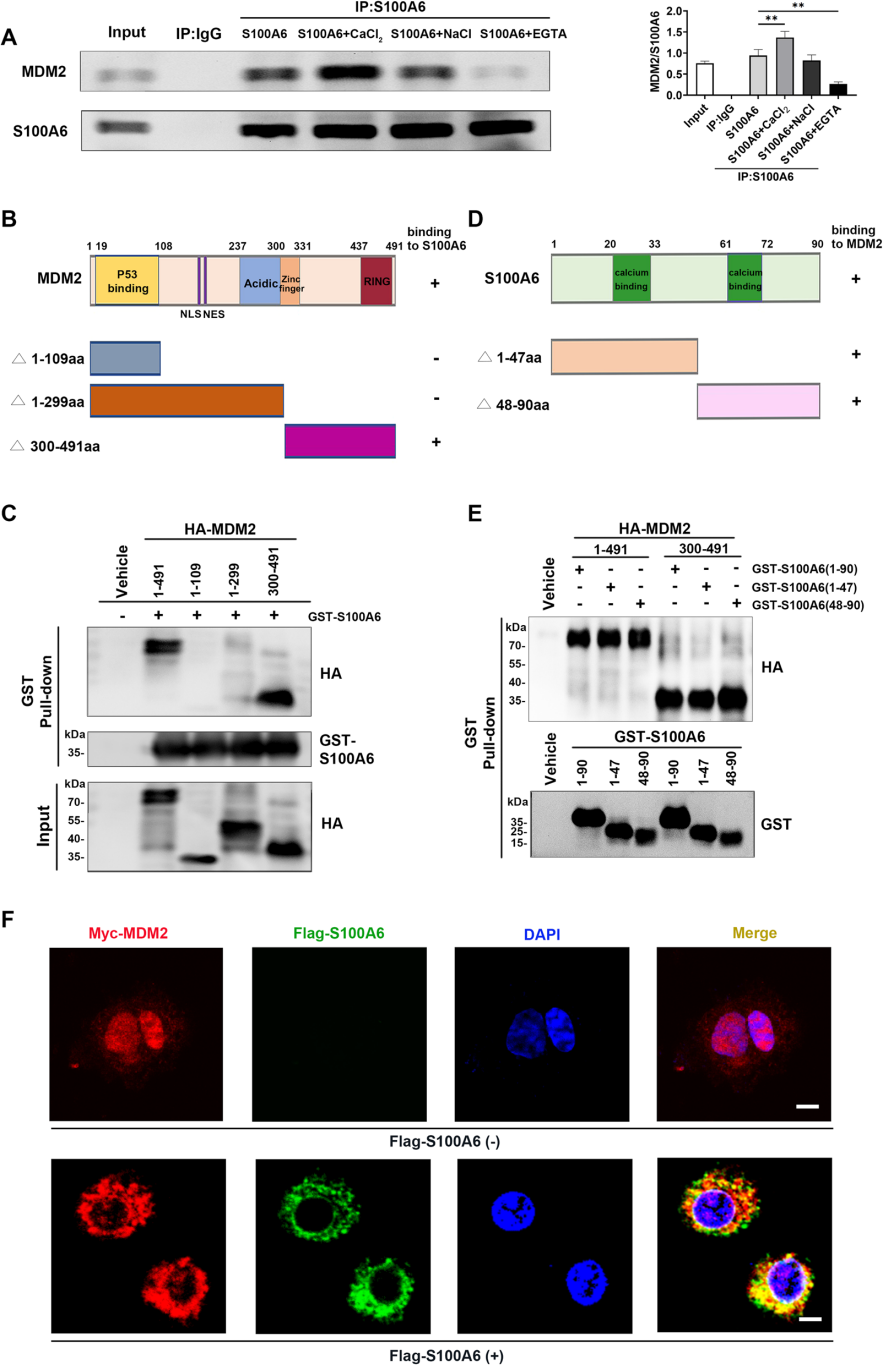
Binding of S100A6 to MDM2 in vivo and in vitro. **A** Co-immunoprecipitation assay for binding between endogenous S100A6 and MDM2 in MCF-7 cells. Cell lysates from MCF-7 were incubated with anti-S100A6 antibody or IgG in the presence of 0.2 M CaCI_2_, 0.2 M NaCl, or 4 mM EGTA. The immunoprecipitates were detected by western blot using an anti-MDM2 antibody. Data from three independent experiments were presented as mean ± SD, which were to compare the effects of CaCI_2_ and EGTA on the combination degree of MDM2 and S100A6 in S100A6-transfected MCF-7 cells. **P < 0.01. **B** A schematic diagram showing different domains of MDM2 and its truncations. **C** GST pull-down assay for binding between GST-S100A6 and HA-tagged full-length MDM2 or its truncations. **D** A schematic diagram showing different domains of S100A6 and its truncations. **E** GST pull-down assay for binding between HA-MDM2 and GST-fused full-length S100A6 or its truncations. **F** Confocal analysis showing the subcellular localization of MDM2 and S100A6. Scale bar: 5 μm

A previous study indicated an interaction between S100A6 and MDM2 by size exclusion chromatography and surface plasmon resonance experiments [20]. Here, co-immunoprecipitation and western blot assays were performed in MCF-7 cells to confirm whether S100A6 could bind to MDM2 in vivo. The result showed that S100A6 could bind to MDM2, and the binding between them was Ca^2+^-dependent, dramatically enhanced by CaCl_2_ (NaCl as a control), and completely inhibited by Ca^2+^ chelator ethylene glycol tetraacetic acid (EGTA) (Fig. 2A).

To screen for the specific domains of S100A6 and MDM2 required for their binding, a series of MDM2 and S100A6 truncations were constructed, and a GST pull-down assay was performed in this study. The MDM2 protein comprises 491 amino acids (aa) and contains several conserved functional domains [22]. The N-terminus is the p53 binding site, which binds to p53 and inhibits p53-mediated transcription. Sequences further toward the central domain are the nuclear localization sequence (NLS) and the nuclear export sequence (NES), which regulate the shuttling of MDM2 between the nucleus and the cytoplasm. Meanwhile, the central domain of MDM2 includes an acidic region that partially overlaps with the zinc finger structure. The very C-terminus is the RING domain, which enables it to have E3 ubiquitin ligase activity [23]. In this study, three MDM2 truncations were constructed: MDM2Δ1 (aa 1–109), MDM2Δ2 (aa 1–299), and MDM2Δ3 (aa 300–491) (Fig. 2B). GST-S100A6 protein was employed to assess its interaction with HA-MDM2 protein, including full-length MDM2 (1–491) and MDM2 fragments 1–109, 1–299, and 300–491. Western blot with anti-HA antibody showed that the MDM2 fragment 300–491 is necessary for S100A6 binding, and no binding between S100A6 and MDM2 1–109 or 1–299 was detected (Fig. 2C).

S100A6 usually forms dimers, and each monomer contains two EF-hand Ca^2+^-binding domains [24]. Two different S100A6 truncations, EF-hand I (1–47) and EF-hand II (48–90) (Fig. 2D), were constructed in this study to examine their binding to MDM2. Both truncations could bind to HA-MDM2 (Fig. 2E). Therefore, both the N-terminal and C-terminal EF-hands of S100A6 are sufficient for their binding.

In addition, the subcellular localizations of S100A6 and MDM2 were observed by immunofluorescence experiment. Myc-MDM2, as indicated by red fluorescence, was mainly expressed in the nucleus, while Flag-S100A6, as marked by green fluorescence, was predominantly localized in the cytoplasm. Flag-S100A6 transfection promoted the translocation of Myc-MDM2 from the nucleus to the cytoplasm, where interacted with Flag-S100A6. Digital merging of confocal microscopic images of Myc-MDM2 (red fluorescence) and Flag-S100A6 (green fluorescence) depicted considerable colocalization (yellow fluorescence) of these proteins in the cytoplasm (with a PCC of 0.76) (Fig. 2F).

### S100A6 induces self-ubiquitination and degradation of MDM2

**Fig. 3:**
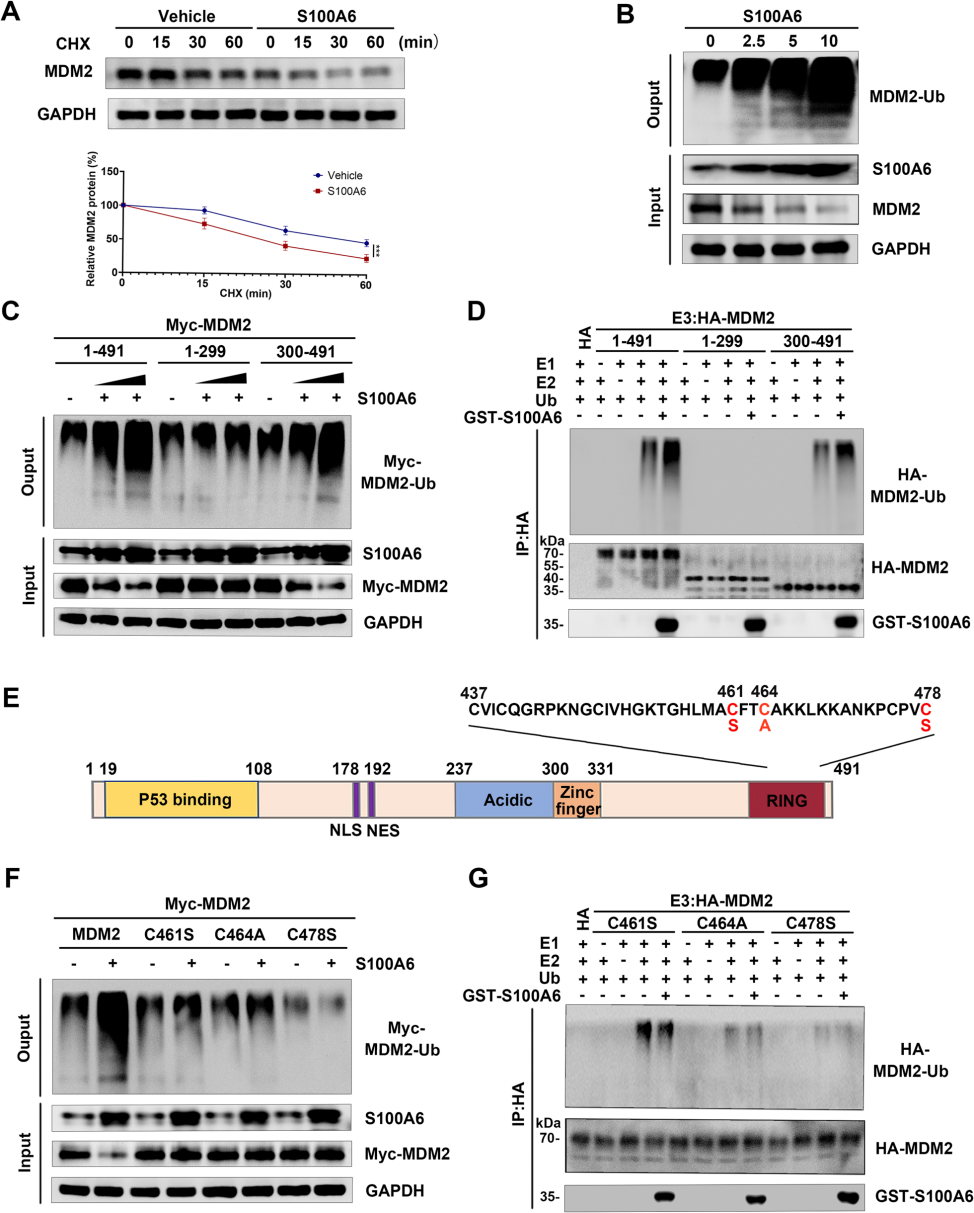
Effects of S100A6 on MDM2 self-ubiquitination and protein degradation. **A** Turnover of MDM2 in MCF-7 cells under transfection of S100A6 (5 μg, S100A6 plasmid) and control plasmid (vehicle) by CHX pulse-chase assay. Curves for relative expression of MDM2 of transfection of S100A6 (red line) and control plasmid (blue line) were shown. Data were shown as mean ± SD; *n* = 3 independent experiments. ***P < 0.001. **B** The effect of S100A6 on MDM2 ubiquitination in MCF-7 cells under transfections of various concentrations of S100A6 in an in vivo ubiquitination assay. **C** The effect of S100A6 (0, 2.5, and 5 μg, S100A6 plasmid) on the ubiquitination of full-length MDM2 or its fragments 1–299 and 300–491 in an in vivo ubiquitination assay. **D** The effect of GST-S100A6 on the ubiquitination of HA-tagged full-length MDM2 or its fragments 1–299 and 300–491 in an in vitro ubiquitination assay. **E** Sequence of the C-terminal RING domain of MDM2 and its mutants C461S, C464A, and C478S. **F** The effect of S100A6 (5 μg, S100A6 plasmid) on the ubiquitination of either wild-type MDM2 or mutant MDM2 C461S, C464A, and C478S in an in vivo ubiquitination assay. **G** The effect of GST-S100A6 on the ubiquitination of HA-MDM2 C461S, C464A, and C478S in an in vitro ubiquitination assay

In addition, this study investigated how MDM2 was regulated by S100A6. It was found that MDM2 degradation was induced by S100A6 at the post-translational level, which was obtained based on the CHX pulse-chase. MDM2 in control cells showed a half-life of 60 min, while S100A6-transfected cells exhibited a half-life of 30 min, which was shortened greatly (Fig. 3A).

Since MDM2 is an E3 ubiquitin ligase and can be ubiquitinated in an autocatalytic manner, the possibility that S100A6 induces MDM2 degradation by a ubiquitination pathway was studied. S100A6 transfection was performed in MCF-7 cells followed by immunoprecipitation and western blot assay. As expected, S100A6 induced MDM2 ubiquitination, and elevated S100A6 led to the enhancement of MDM2 ubiquitination in a dose-dependent manner (Fig. 3B).

An in vivo ubiquitination assay was performed in MCF-7 cells by co-transfection of Ub, S100A6, and full-length MDM2 (1–491), the MDM2 fragments 1–299, or 300–491 tagged with Myc. Enforced expression of S100A6 increased the ubiquitination of full-length MDM2 (1–491) and MDM2 fragment 300–491. However, S100A6 did not increase the ubiquitination of MDM2 1–299 (an MDM2 fragment with a deletion of the C-terminal domain and loss of S100A6 binding activity) (Fig. 3C).

Furthermore, GST-S100A6 and HA-MDM2, including full-length MDM2 (1–491) and MDM2 fragments 1–299 and 300–491, were expressed in *E. coli* to assess the capacity of S100A6 to induce the ubiquitination of MDM2 in vitro. The full-length MDM2 (1–491) and MDM2 fragment 300–491, but not MDM2 1–299, underwent ubiquitination by GST-S100A6 in vitro (Fig. 3D). Therefore, binding to the MDM2 fragment 300–491 is essential for S100A6 to induce MDM2 ubiquitination.

To further confirm that S100A6 induced the self-ubiquitination of MDM2, the RING finger mutated MDM2 C461S, C464A, and C478S, which lose E3 activity, were constructed here (Fig. 3E). The results from the in vivo ubiquitination assay showed that S100A6 failed to induce ubiquitination of MDM2 C461S, C464A, and C478S (Fig. 3F). As also shown in the in vitro ubiquitination assay, the recombinant GST-S100A6 failed to increase the ubiquitination of HA-MDM2 C461S, C464A, and C478S when it was incubated in ubiquitin reaction buffer (Fig. 3G). Therefore, S100A6 induces MDM2 self-ubiquitination and its corresponding degradation.

### S100A6 increases MDM2 self-ubiquitination by disrupting MDM2–HAUSP–DAXX interactions

**Fig. 4:**
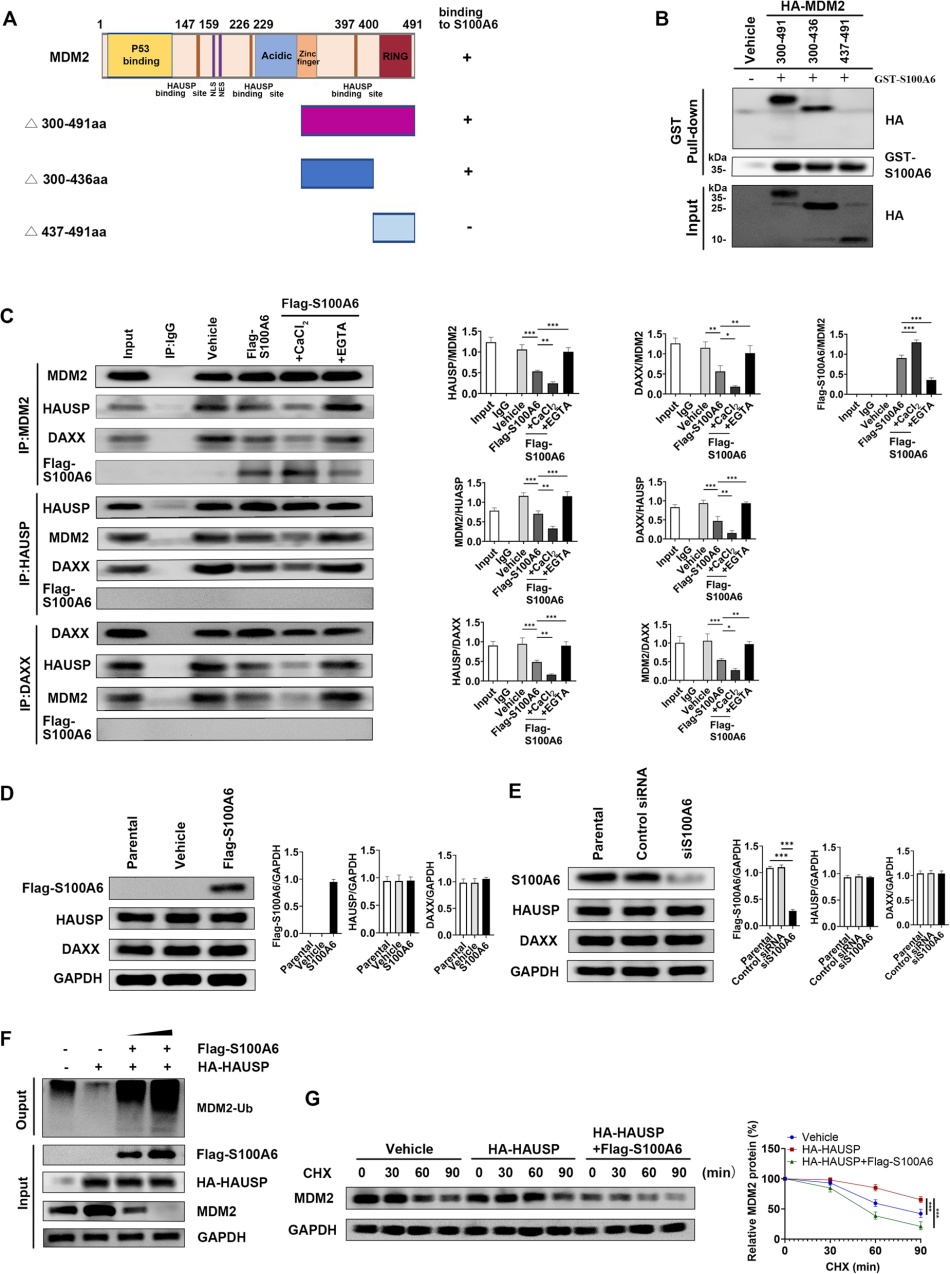
S100A6 increases MDM2 self-ubiquitination by disrupting MDM2–HAUSP–DAXX interactions. **A** A schematic diagram showing different domains of MDM2 and its truncations. **B** GST pull-down assay for binding between GST-S100A6 and HA-MDM2 300–491, 300–436, and 437–491. **C** Co-immunoprecipitation assay for MDM2–HAUSP–DAXX interactions and binding between S100A6 and MDM2, HAUSP, or DAXX in MCF-7 cells in the presence of CaCl_2_ or EGTA. **D** Expressions of HAUSP and DAXX in MCF-7 cells transfected with S100A6 (5 μg, S100A6 plasmid). **E** Expressions of HAUSP and DAXX in MCF-7 transfected with S100A6 siRNA (5 μg, siS100A6). Histograms showed the densitometric analyses of indicated proteins. Data were shown as mean ± SD; *n* = 3 independent experiments. *P < 0.05, **P < 0.01, and ***P < 0.001 **F** In vivo ubiquitination assay for testing the effect of HAUSP with or without S100A6 (2.5 and 5 μg, S100A6 plasmid) on MDM2 ubiquitination in MCF-7 cells. **G** CHX pulse-chase assay for testing the effect of HAUSP with or without S100A6 on MDM2 turnover. Curves for relative expression of MDM2 transfected with control plasmid (blue line), HA-HAUSP plasmid (red line), and HA-HAUSP and Flag-S100A6 plasmid (green line). Data were shown as mean ± SD; *n* = 3 independent experiments. *P < 0.05, **P < 0.01, and ***P < 0.001

In further mechanistic studies, it was found that S100A6 did not bind to the RING domain of MDM2 (437–491) but to the 300–436 fragment containing the binding site of the deubiquitinating enzyme HAUSP (Fig. 4A, B). Earlier studies demonstrated that the MDM2 ubiquitination was regulated by HAUSP [25]. MDM2, HAUSP, and DAXX interact with each other to form a tertiary complex that reduces the self-ubiquitination of MDM2. Therefore, this finding suggested that S100A6 might affect the formation of the MDM2–HAUSP–DAXX complex, thus preventing HAUSP from stabilizing MDM2. Co-immunoprecipitation assays showed that S100A6 interfered with MDM2–HAUSP–DAXX interactions. The binding between MDM2 and HAUSP or DAXX and that between HAUSP and DAXX were decreased in S100A6-transfected cells (Fig. 4C). Likewise, this effect was enhanced by CaCl_2_ and inhibited by EGTA. Additionally, there was no binding between S100A6 and HAUSP or DAXX (Fig. 4C), nor did S100A6 alter the expressions of HAUSP and DAXX (Fig. 4D, E).

In vivo ubiquitination assay and CHX pulse-chase assay were then performed by transfection of HAUSP and S100A6 to investigate the function of S100A6 in HAUSP-mediated MDM2 deubiquitination and stabilization. Enforced expression of HAUSP decreased the MDM2 ubiquitination. However, exogenous S100A6 enhanced the MDM2 ubiquitination even in the presence of enforced expression of HAUSP (Fig. 4F). HAUSP stabilized MDM2, but S100A6 decreased MDM2 stability. Compared with the control cells, MDM2 showed a prolonged half-life in HAUSP-transfected cells, but the half-life of which was shortened in HAUSP and S100A6 co-transfected cells (Fig. 4G). Taken together, these results indicate that S100A6 binds to the binding site of HAUSP in MDM2 and disrupts MDM2–HAUSP–DAXX interactions, thereby enhancing the MDM2 self-ubiquitination.

### S100A6 suppresses breast cancer cell growth and enhances the sensitivity of cancer cells to paclitaxel-induced apoptosis and cell-cycle arrest in a manner dependent on MDM2 inhibition

**Fig. 5:**
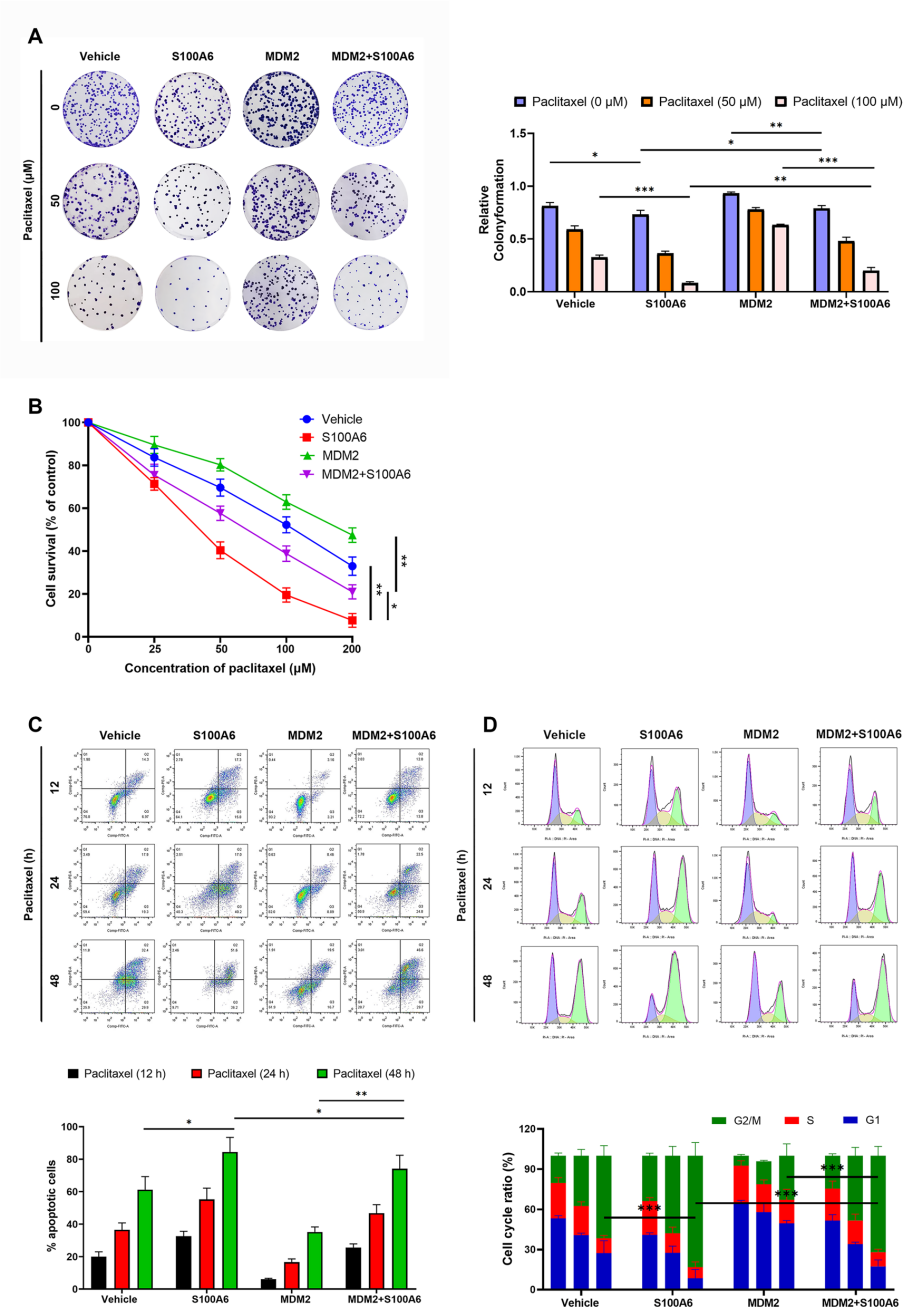
Effects of S100A6 on the growth, apoptosis, and cell cycle of breast cancer cells. **A** Clonogenic assay of MCF-7 cells transfected with S100A6, MDM2, or MDM2 and S100A6 with or without paclitaxel. **B** WST-1 results of MCF-7 transfected with S100A6, MDM2, or MDM2 and S100A6, and those of the cells after 24 h of paclitaxel treatment. **C** and **D** Cell apoptosis and cell-cycle results of MCF-7 cells transfected with S100A6, MDM2, or MDM2 and S100A6, and those of the cancer cells treated with 100 μM paclitaxel for different times, respectively. Data were shown as mean ± SD; *n* = 3 independent experiments. *P < 0.05, **P < 0.01, and ***P < 0.001

Next, whether S100A6 could suppress the growth of breast cancer cells and enhance their sensitivity to chemotherapy was investigated. A clonogenic assay was performed in MCF-7 cells transfected with S100A6, MDM2, or MDM2 and S100A6, which were allowed to react with paclitaxel at various dosages. Without paclitaxel, S100A6 suppressed the clonogenicity of cancer cells, and colony formation was suppressed in S100A6-transfected cells compared with control-transfected cells. With paclitaxel, S100A6 enhanced the sensitivity of cancer cells to paclitaxel-induced colony formation suppression, and suppression of colony formation in S100A6-transfected cells was more obvious than that in control-transfected cells after treatment with paclitaxel. However, the suppression of colony formation in MDM2 and S100A6 co-transfected cells was less than that in S100A6-transfected cells. Therefore, the effect of S100A6 is dependent on MDM2 inhibition (Fig. 5A).

In addition, the cytotoxic, apoptotic, and cell-cycle arrested effects of paclitaxel on S100A6-transfected cells were measured. The results exhibited that the sensitivity of cancer cells to paclitaxel-induced cytotoxicity, apoptosis, and cell-cycle arrest was enhanced obviously by S100A6, which is similar to the growth inhibitory activity. The WST-1 (Fig. 5B), cell apoptosis (Fig. 5C), and cell-cycle assays (Fig. 5D) suggested that S100A6-transfected cells were more responsive to paclitaxel-induced cytotoxicity, apoptosis, and G2/M phase arrest than the control-transfected cells. Likewise, the effect of S100A6 is dependent on its inhibition on MDM2. Paclitaxel showed less cytotoxic, apoptotic, and G2/M phase arrested effects on MDM2 and S100A6 co-transfected cells than on S100A6-transfected cells.

### S100A6 suppresses tumor growth and enhances sensitivity to paclitaxel in a xenograft model of breast cancer

**Fig. 6:**
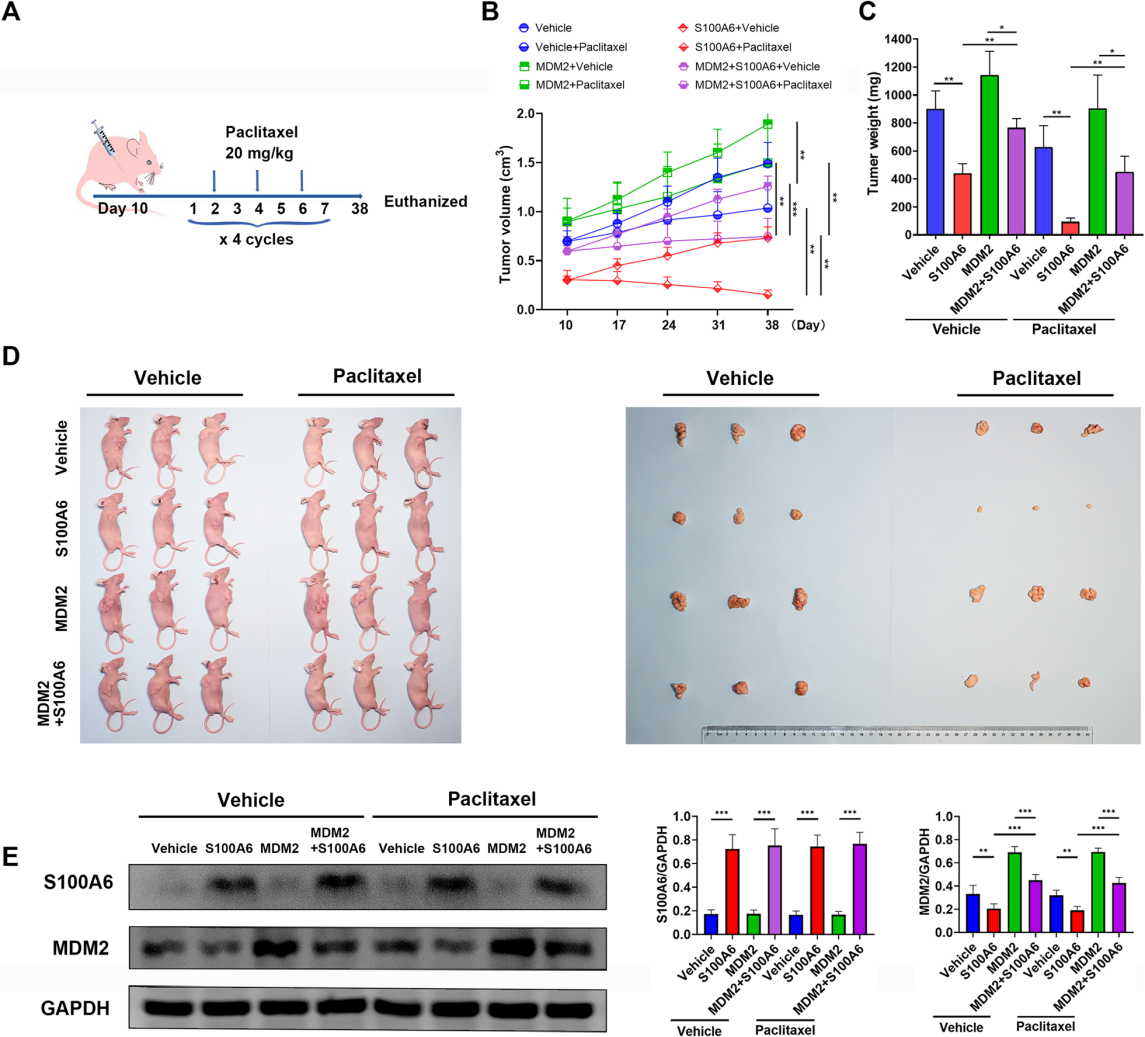
S100A6 suppresses tumor growth and enhances sensitivity to chemotherapy in xenograft model of breast cancer. **A** Schematic diagram of subcutaneous xenografts and schedule of paclitaxel administration. **B** Size of xenograft tumor. **C** Weight of xenograft tumor on day 38. **D** Typical pictures from each group. **E** Expressions of S100A6 and MDM2 in tumor tissues. Data were shown as mean ± SD; *n* = 3 independent experiments. *P < 0.05, **P < 0.01, and ***P < 0.001

Given the in vitro observations, suppression of S100A6 on tumor development and its enhancement on sensitivity to paclitaxel were investigated in a nude mouse xenograft model. For the experiment, xenografts in nude mice were established by inoculating MCF-7 cells transfected with S100A6, MDM2, or MDM2 and S100A6. Mice were then treated by intraperitoneal injection of paclitaxel (20 mg/kg, 3 times/week, for 4 weeks) (Fig. 6A). Tumor growth was observed during the entire treatment. It was found that S100A6 suppressed the tumor growth and enhanced its sensitivity to paclitaxel. Regardless of paclitaxel treatment, the tumor growth for mice with S100A6-transfected cells was obviously delayed in contrast to that with control-transfected cells. Likewise, the effect of S100A6 was dependent on its inhibition on MDM2. Overexpressed MDM2 attenuated tumor growth suppression by S100A6. There was less suppression of tumor growth in mice with MDM2 and S100A6 co-transfected cells than in those with S100A6-transfected cells (Fig. 6B–D). Finally, the removed tumor tissue was subjected to western blot to verify the expressions of S100A6 and MDM2. Expression of MDM2 was significantly inhibited in the xenografts from S100A6-transfected cells. However, inhibition of MDM2 in xenografts from MDM2 and S100A6 co-transfected cells was weaker than that in the xenografts from S100A6-transfected cells (Fig. 6E).

### S100A6 is a predictive biomarker for the efficacy of neoadjuvant chemotherapy in breast cancer patients

**Fig. 7:**
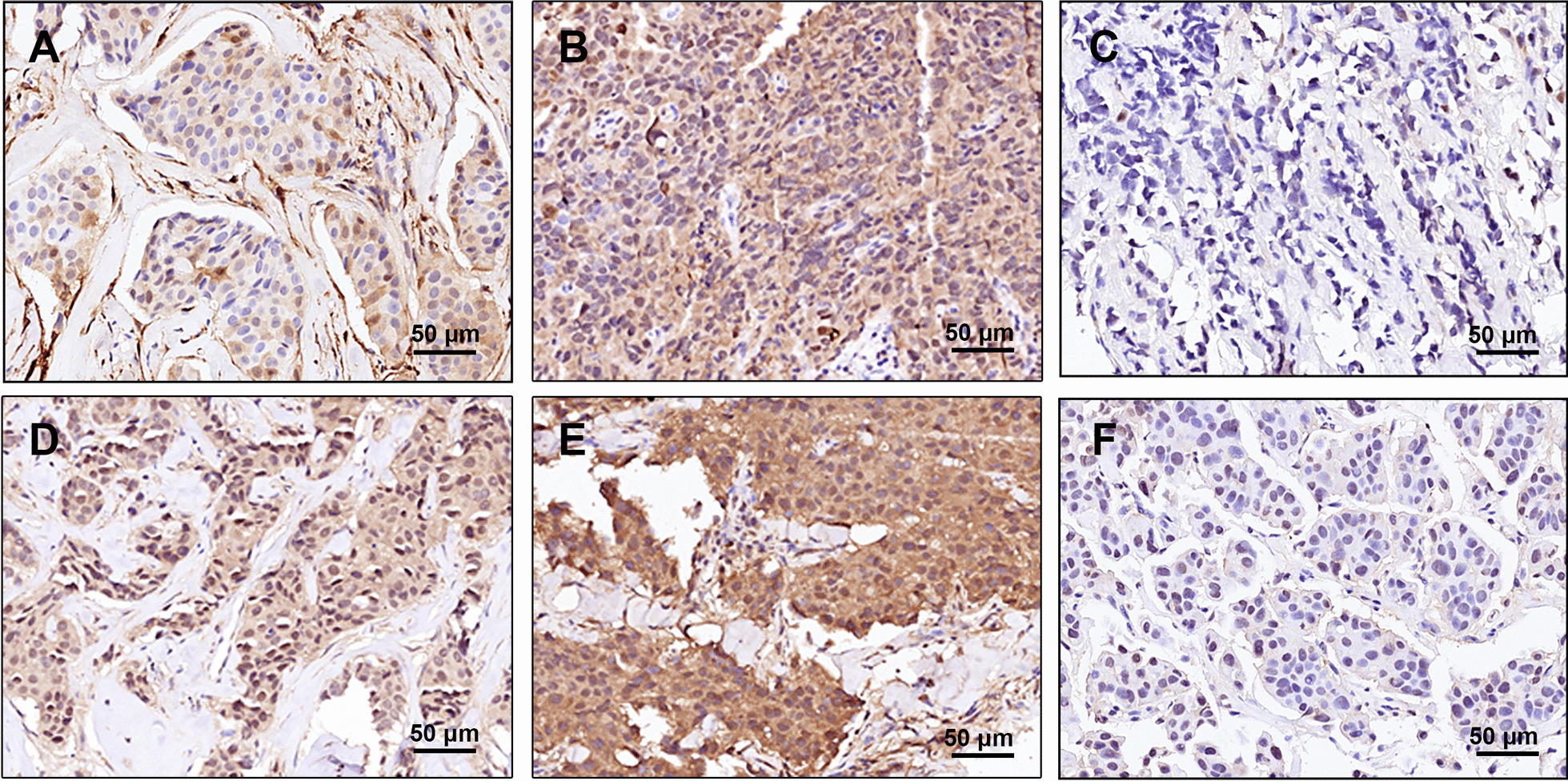
Representative images of immunohistochemical staining for S100A6 and MDM2. **A**–**C** S100A6 low staining, high staining, and negative staining in invasive breast cancer, respectively. **D**–**F** MDM2 low staining, high staining, and negative staining in invasive breast cancer, respectively

To assess the clinical relevance between S100A6 and MDM2 in breast cancer patients, especially that between the expression of S100A6 and chemotherapy efficacy, immunohistochemistry was performed on tumor tissues in a cohort of 107 invasive breast cancer patients receiving neoadjuvant chemotherapy with epirubicin and cyclophosphamide followed by docetaxel (EC-T). Among them, 55 cases (51.4%) showed high immunoreactivity for S100A6, while 52 cases (48.6%) expressed low immunoreactivity. The Chi-square test revealed that S100A6 was negatively correlated with expression of MDM2 (*P* < 0.0001) (Table 1). Representative images for expressions of S100A6 and MDM2 are presented in Fig. 7.

**Table 1:** Correlation between the expressions of S100A6 and MDM2 in invasive breast cancer

Variable	Total (*n* = 107)	S100A6 high, no. (%)	S100A6 low, no. (%)	*P* value	MDM2 high, no. (%)	MDM2 low, no. (%)	*P* value
*S100A6 expression*							
High	55				8 (14.5)	47 (85.5)	< 0.0001
Low	52				31 (59.6)	21 (40.4)	
*Molecular subtype*							
Luminal (non-HER2 +)	57	28 (49.1)	29 (50.9)	0.834	21 (36.8)	36 (63.2)	0.982
HER2 +	23	13 (56.5)	10 (43.5)		8 (34.8)	15 (65.2)	
TNBC	27	14 (51.9)	13 (48.1)		10 (37.0)	17 (63.0)

Furthermore, the correlation between the expression of S100A6 and the efficacy of neoadjuvant chemotherapy (EC-T) was investigated. It was found that pCR in patients treated with preoperative chemotherapy (EC-T) was significantly correlated with the expression of S100A6. Specifically, patients with highly expressed S100A6 possessed a significantly higher rate of pCR (*P* < 0.0001) (Table 2). Univariate and multivariate analyses suggested that high expression of S100A6 was an independent factor in predicting pCR (*P* < 0.0001 and *P* = 0.010, respectively) (Table 3).

**Table 2:** Correlation between chemoresponse and expression of S100A6 in invasive breast cancer

Subtypes	n	pCR rate (S100A6 high/low)	*P* value
All patients	107	41.8%/11.5%	< 0.0001
Luminal (non-HER2 +)	57	17.9%/3.4%	0.102
HER2 +	23	69.2%/20%	0.036
TNBC	27	64.3%/23.1%	0.031

**Table 3:** Univariate and multivariate analyses of pCR against various characteristics in breast cancer patients who received neoadjuvant chemotherapy

Variable	pCR rate (%)	Univariate analysis *P* value	Multivariate analysis *P* value
S100A6 (high/low)	41.8%/11.5%	< 0.0001	0.010
MDM2 (high/low)	15.4%/33.8%	< 0.0001	0.215
Size (pT1-2/pT3-4)	27.2%/27.0%	0.680	0.757
Histological grade (I–II/III)	24.7%/33.3%	0.300	0.543
LN metastasis (posi/neg)	27.7%/25.0%	0.882	0.679
MIB (< 10%/ ≥ 10%)	12.5%/29.7%	0.177	0.664
ERα (posi/neg)	19.1%/38.6%	0.045	0.798
PR (posi/neg)	21.3%/34.8%	0.188	0.294
Her2 (posi/neg)	47.8%/21.4%	0.008	0.001
Molecular subtype (TNBC/others)	44.4%/21.3%	0.046	0.011

## Discussion

**Fig. 8:**
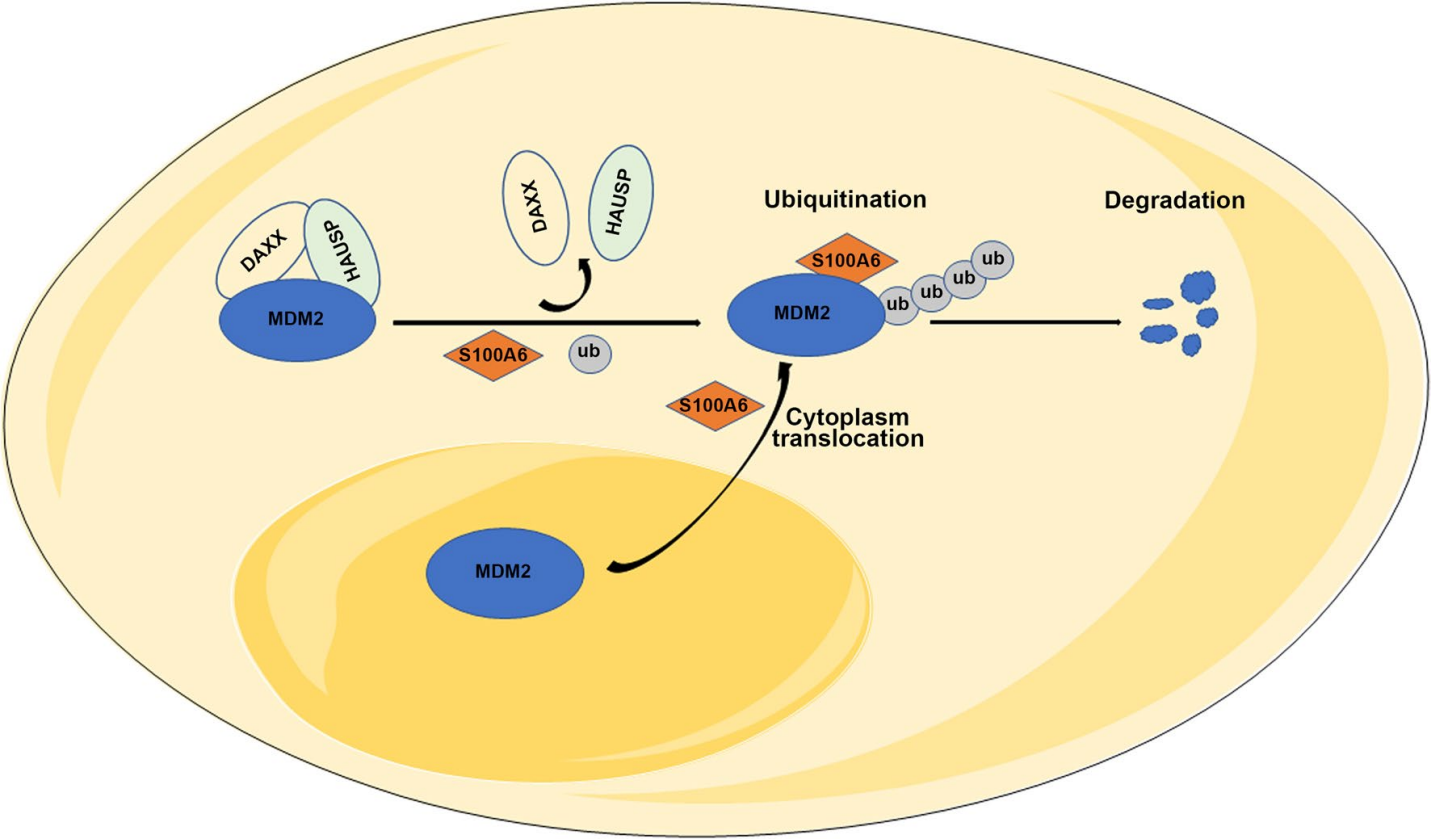
Proposed model for the regulation of MDM2 by S100A6. S100A6 promotes the translocation of MDM2 from the nucleus to the cytoplasm. In the cytoplasm, S100A6 binds to the binding site of HAUSP in MDM2, disrupting the MDM2–HAUSP–DAXX interactions and inducing the MDM2 self-ubiquitination and degradation, thereby suppressing the breast cancer growth and enhancing its sensitivity to chemotherapy

In the present study, it was found that S100A6 bound to MDM2 and exerted an induction effect on MDM2 self-ubiquitination and degradation. It was also found that by disrupting the MDM2–HAUSP–DAXX complex, S100A6 induced MDM2 self-ubiquitination. Meanwhile, S100A6 also recruited MDM2 from the nucleus to the cytoplasm (Fig. 8). Importantly, the S100A6-mediated MDM2 degradation was demonstrated to be of clinical importance. In both breast cancer cells and xenograft model, S100A6 suppressed the tumor growth and enhanced its sensitivity to paclitaxel, which were dependent on MDM2 inhibition. Furthermore, after treatment with EC-T neoadjuvant chemotherapy, patients with high expression of S100A6 exhibited a higher rate of pCR.

The co-immunoprecipitation first demonstrated that S100A6 and MDM2 could interact with each other in breast cancer cells. A further observation from the GST pull-down assay showed that S100A6 specifically bound to a fragment (300–436) close to the C-terminal RING domain of MDM2, which was not in line with the finding of a previous study using size exclusion chromatography and surface plasmon resonance. This study showed that S100A6 bound to the N-terminal domain of MDM2 [20]. It was believed that the reason for the different results may be that they performed physical experiments and did not perform an in-depth analysis on the binding of S100A6 and MDM2 in the overall cellular environment.

MDM2 is not stable so it can be ubiquitinated and degraded by autocatalysis [26]. However, as an oncoprotein, MDM2 increases its protein stability by binding to many other cellular molecules [5]. For example, MDM2 becomes stabilized when binding to HAUSP and DAXX to form a tertiary complex, which is not conductive to MDM2 self-ubiquitination [27]. It was demonstrated in this study that S100A6 disrupted MDM2–HAUSP–DAXX interactions and increased the MDM2 ubiquitination and degradation. Regarding how S100A6 disrupted the MDM2–HAUSP–DAXX complex, this study showed that S100A6 bound to MDM2 300–436, which contained the HAUSP binding site. Therefore, S100A6 may compete with HAUSP for binding to MDM2 and dissociate DAXX from MDM2, thereby preventing the MDM2–HAUSP–DAXX complex from stabilizing the MDM2. This is an important finding of this study, which determines the unique mechanism by which S100A6 promotes MDM2 protein degradation.

In addition, overexpressed S100A6 promoted MDM2 shuttling from the nucleus to the cytoplasm and increased its colocalization with S100A6, although the mechanism remains unknown. S100A6 is predominantly located in the cytoplasm [28], so S100A6 may not directly carry MDM2 out of the nucleus. It is expected based on this study that S100A6 might regulate other signals or molecules that trigger MDM2 translocation. For example, phosphorylation of MDM2 on S166 and S186 by PI3K/Akt promoted MDM2 translocation from the cytoplasm into the nucleus. It is speculated that S100A6, which regulates phosphatase activity [29], may alter the intracellular distribution of MDM2 by dephosphorylating MDM2.

S100A6 is expressed in various cell types of human normal tissues [30, 31]. However, the expression and role of S100A6 in cancer cells have not been extensively studied [32]. It has been reported that S100A6 is over-expressed and associated with disease development and malignant progression in gastric cancer [33], pancreatic cancer [34], and colon cancer [35] but under-expressed in prostate cancer [36] and oral cancer [37]. However, the expression of S100A6 in breast cancer is not yet conclusive. This study examined the expression of S100A6 in different breast cancer cell lines and focused on the role of S100A6 in the progression and chemotherapy of breast cancer. In vitro, S100A6 inhibited the clonogenicity of breast cancer cells and enhanced their sensitivity to paclitaxel-induced cytotoxicity, apoptosis, and G2/M phase arrest. In vivo, S100A6 also suppressed the tumor growth and enhanced its sensitivity to paclitaxel in a nude mouse xenograft model. This study revealed that S100A6 was highly expressed in 51% of invasive breast cancer patients (among 107 cases).

Neoadjuvant chemotherapy promotes the disease-free survival and overall survival rates in combined therapy for breast cancer patients. Meanwhile, it enables more patients to undergo the breast-conserving surgery and significantly improves their quality of life without elevating the rate of local recurrence [38]. As a well-tolerated microtubule stabilizer [39], paclitaxel has become a first-line chemotherapeutic drug for neoadjuvant chemotherapy of breast cancer, especially for advanced metastatic breast cancer [40]. However, its efficacy is restricted by the resistance inevitably acquired after long-term exposure [41]. MDM2 is proved to be associated with chemoresistance in many tumors, including breast cancer. It is reported that MDM2 is upregulated in paclitaxel resistant cells, and its inhibition re-sensitizes the resistant cells to paclitaxel [42, 43]. MDM2 inhibitors in combination with chemotherapy in patients with advanced, metastatic, or unresectable solid tumors have been studied clinically [44]. It was found that high expression of S100A6 in patients receiving EC-T neoadjuvant chemotherapy had a significantly higher rate of pCR, which was more significant for HER2 + and TNBC patients (especially TNBC patients) who were predominantly treated by neoadjuvant chemotherapy. And, high expression of S100A6 was determined to be an independent factor in predicting pCR, suggesting that S100A6 was a predictive biomarker for efficacy of neoadjuvant chemotherapy in breast cancer patients. Therefore, elevating the expression level of S100A6 in breast cancer cells can not only promote the degradation of the oncoprotein MDM2 and inactivate some cancer progression pathways, but also lower the occurrence of chemoresistance, achieving better therapeutic effects.

In conclusion, this study demonstrates a novel and important role of S100A6 in the negative regulation of MDM2. By disrupting the MDM2–HAUSP–DAXX-HAUSP-DAXX complex, S100A6 induces MDM2 self-ubiquitination and its degradation, which suppresses the growth of breast cancer and enhances its sensitivity to chemotherapy. These results suggest that S100A6 can be a predictive marker for chemotherapy response and that it may be a significant means to chemotherapy by elevating the expression level of S100A6 in patients with highly expressed MDM2 (Additional file. 1).

## Supplementary Information

**Additional file 1.** Raw data for western blot and colony formation assay.

## Data Availability

Data were generated by the authors and available on request.
