# Supplementary material for: S100A6 inhibits MDM2 to suppress breast cancer growth and enhance sensitivity to chemotherapy
Source: Breast Cancer Res. 2023 May 22;25:55. doi: 10.1186/s13058-023-01657-w (PMC10204293; doi:10.1186/s13058-023-01657-w)

S100A6 inhibits MDM2 to suppress breast cancer growth and enhance sensitivity to chemotherapy

Mengxin Qi, Xianglan Yi, Baohui Yue, Mingxiang Huang, Sheng Zhou * and Jing Xiong *
Institute of Pathology, Tongji Hospital, Tongji Medical College, Huazhong University of Science and Technology, Wuhan, China

FIGURE 1

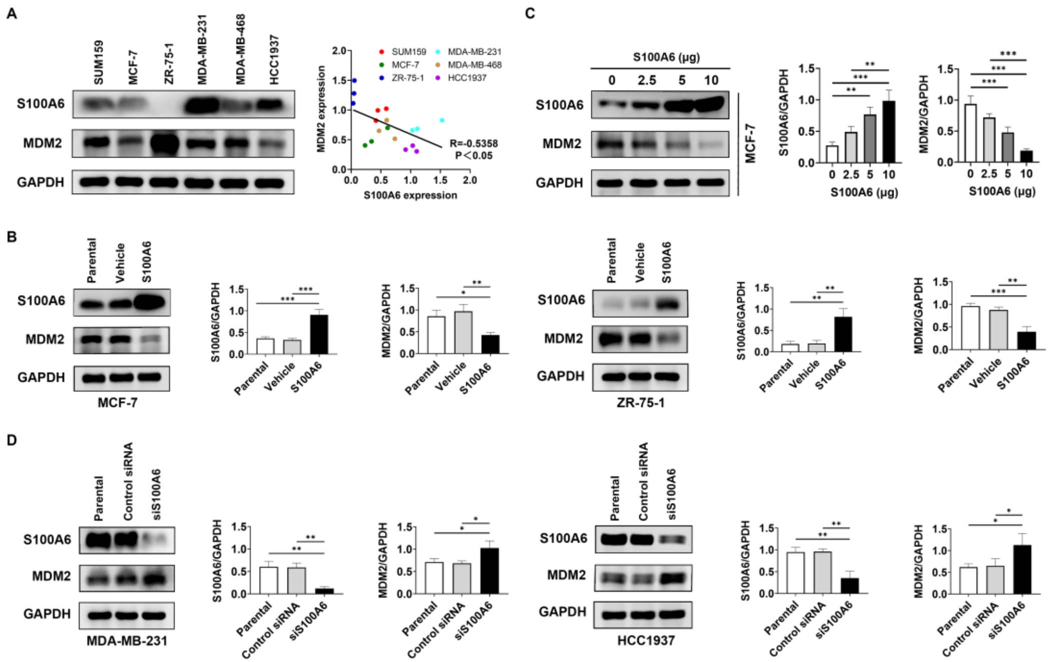
A
SUM159
MCF-7
ZR-75-1
MDA-MB-231
MDA-MB-468
HCC1937
S100A6
MDM2
GAPDH
MDM2 expression
S100A6 expression
R=-0.5358
P＜0.05
C
S100A6 (μg)
0
2.5
5
10
MCF-7
S100A6/GAPDH
MDM2/GAPDH
B
Parental
Vehicle
S100A6
MCF-7
ZR-75-1
D
Parental
Control siRNA
siS100A6
MDA-MB-231
HCC1937

A

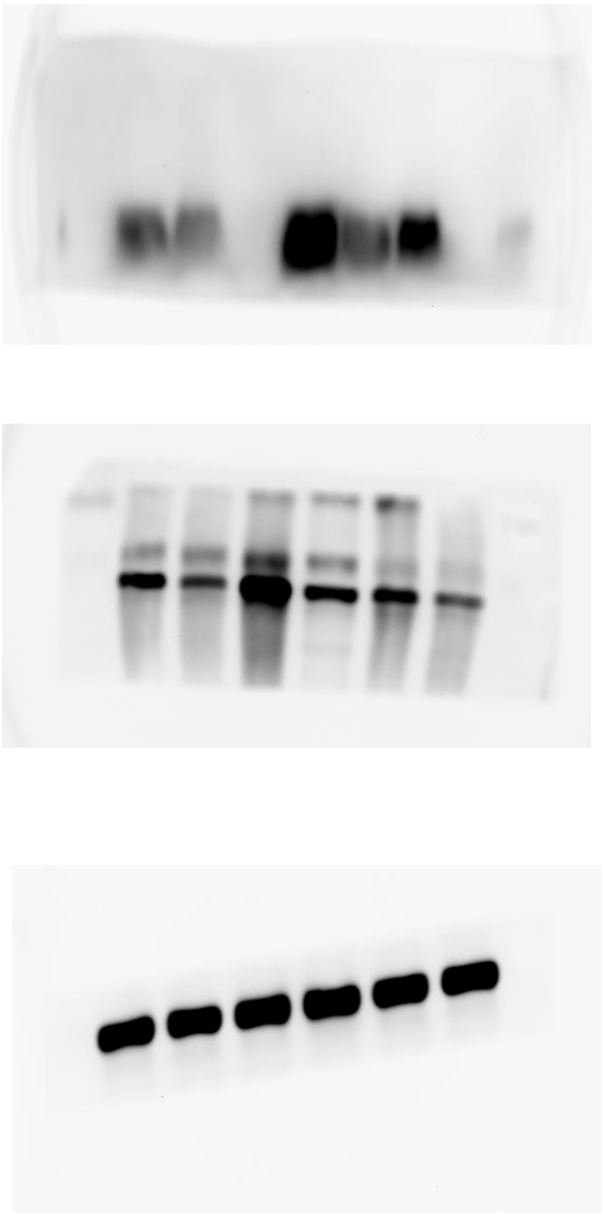

C

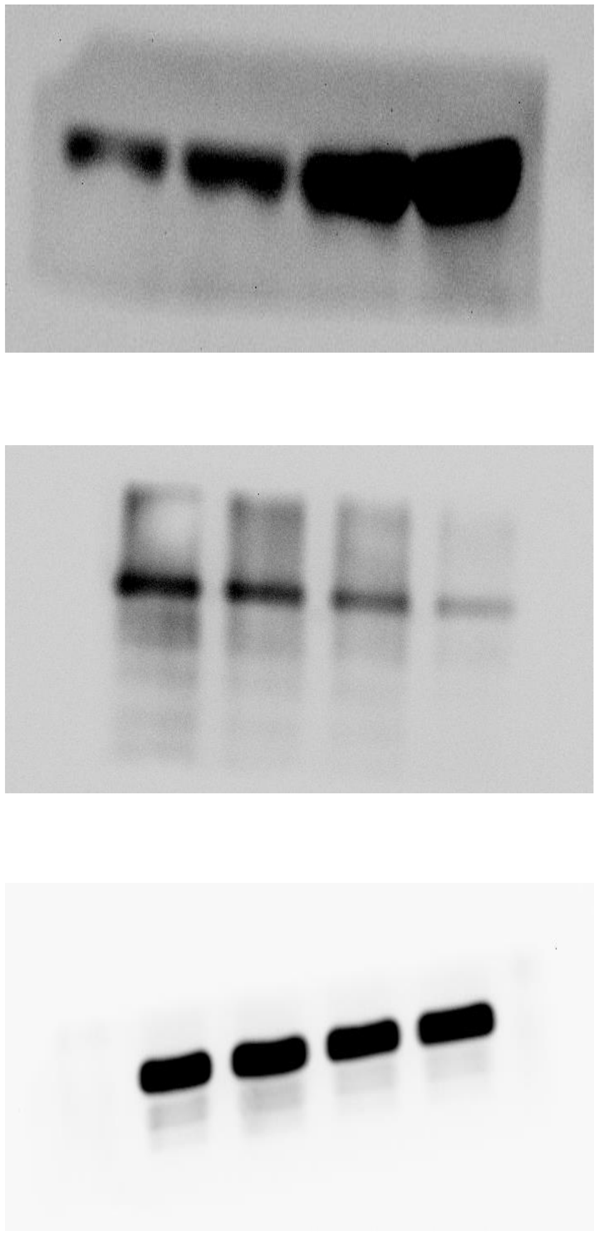

FIGURE 1

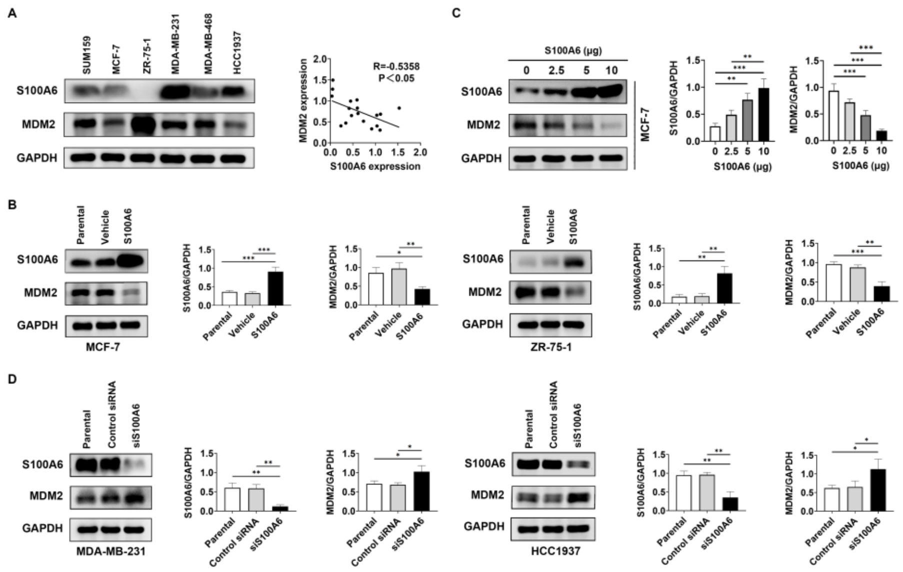
A
SUM159
MCF-7
ZR-75-1
MDA-MB-231
MDA-MB-468
HCC1937
S100A6
MDM2
GAPDH
R=-0.5358
P＜0.05
MDM2 expression
S100A6 expression
B
Parental
Vehicle
S100A6
MCF-7
ZR-75-1
S100A6/GAPDH
MDM2/GAPDH
C
S100A6 (μg)
0
2.5
5
10
MCF-7
D
Parental
Control siRNA
siS100A6
MDA-MB-231
HCC1937

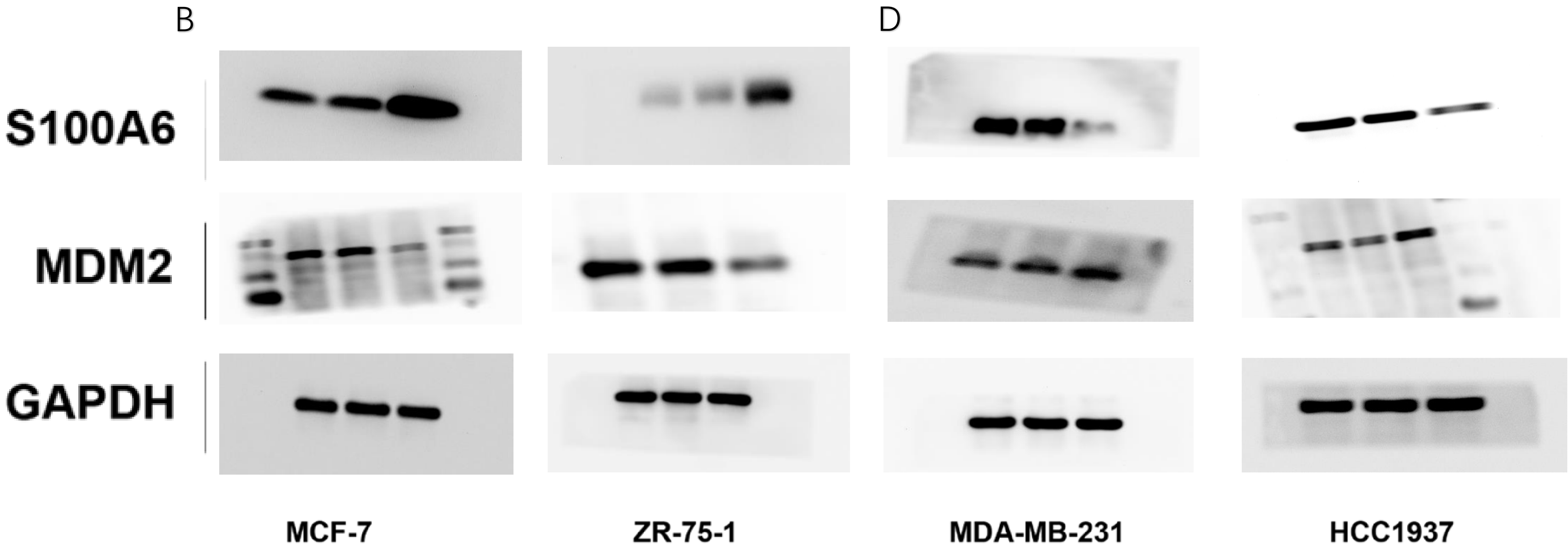
B
D
S100A6
MDM2
GAPDH
MCF-7
ZR-75-1
MDA-MB-231
HCC1937

FIGURE 2

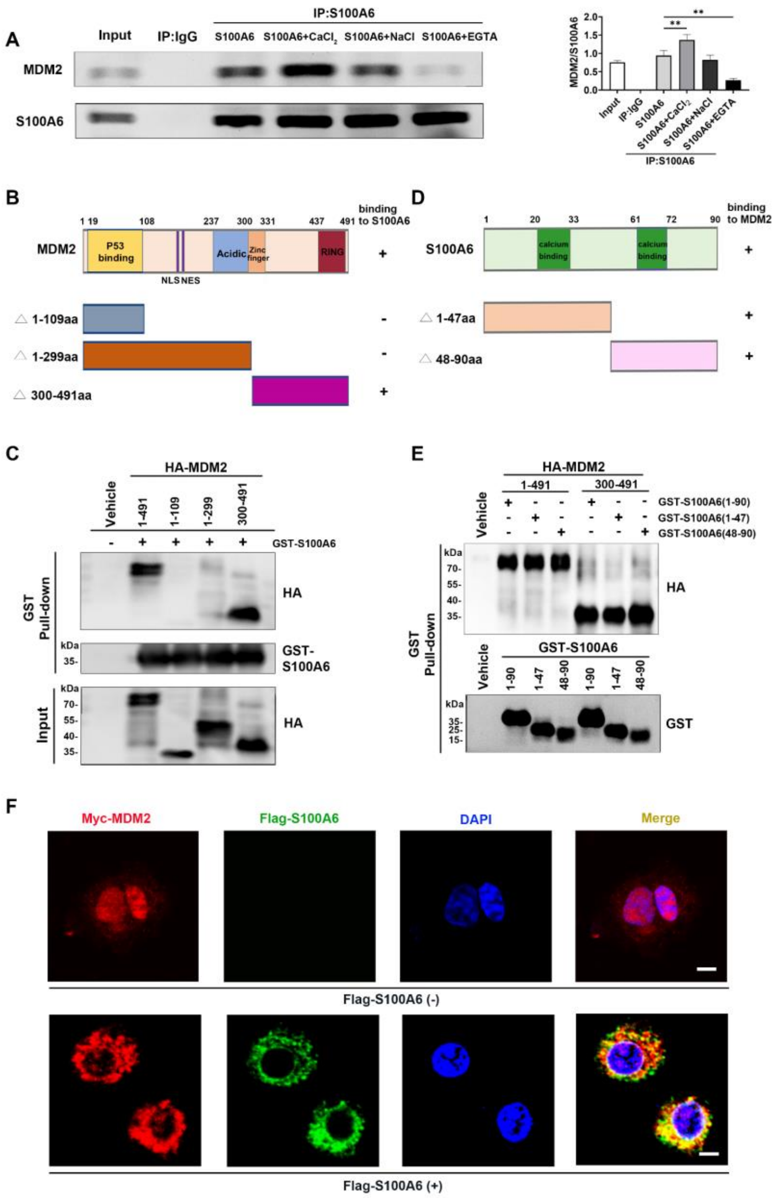
A
Input
IP:IgG
IP:S100A6
S100A6
S100A6+CaCl2
S100A6+NaCl
S100A6+EGTA
MDM2
S100A6
MDM2/S100A6
**
**
B
binding to S100A6
MDM2
P53 binding
Acidic
Zinc finger
RING
NLS NES
△ 1-109aa
△ 1-299aa
△ 300-491aa
D
binding to MDM2
S100A6
calcium binding
△ 1-47aa
△ 48-90aa
C
HA-MDM2
Vehicle
1-491
1-109
1-299
300-491
GST-S100A6
GST Pull-down
HA
GST-S100A6
Input
kDa
E
HA-MDM2
1-491
300-491
GST-S100A6(1-90)
GST-S100A6(1-47)
GST-S100A6(48-90)
GST
F
Myc-MDM2
Flag-S100A6
DAPI
Merge
Flag-S100A6 (-)
Flag-S100A6 (+)

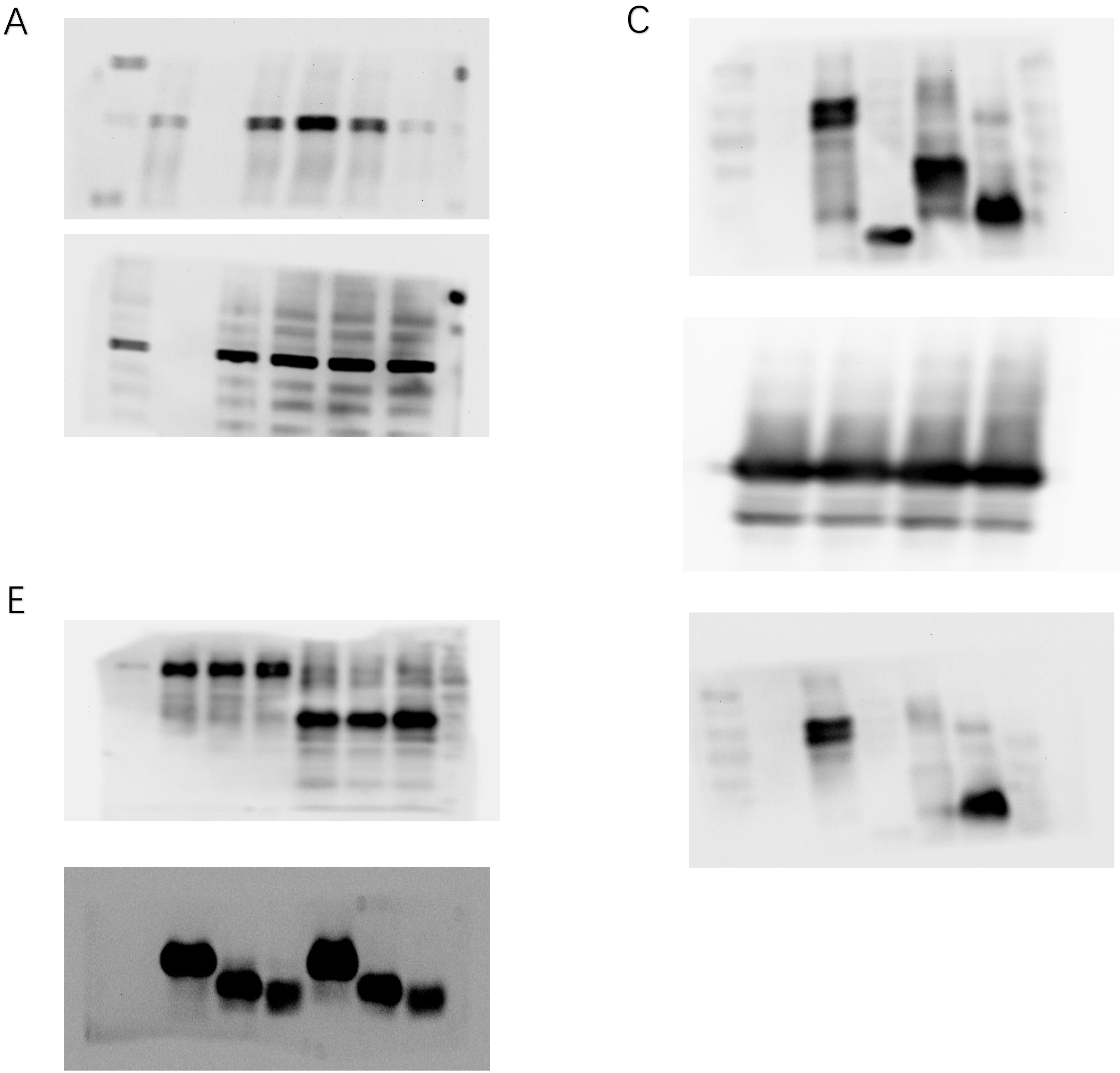
A
C
E

FIGURE 3

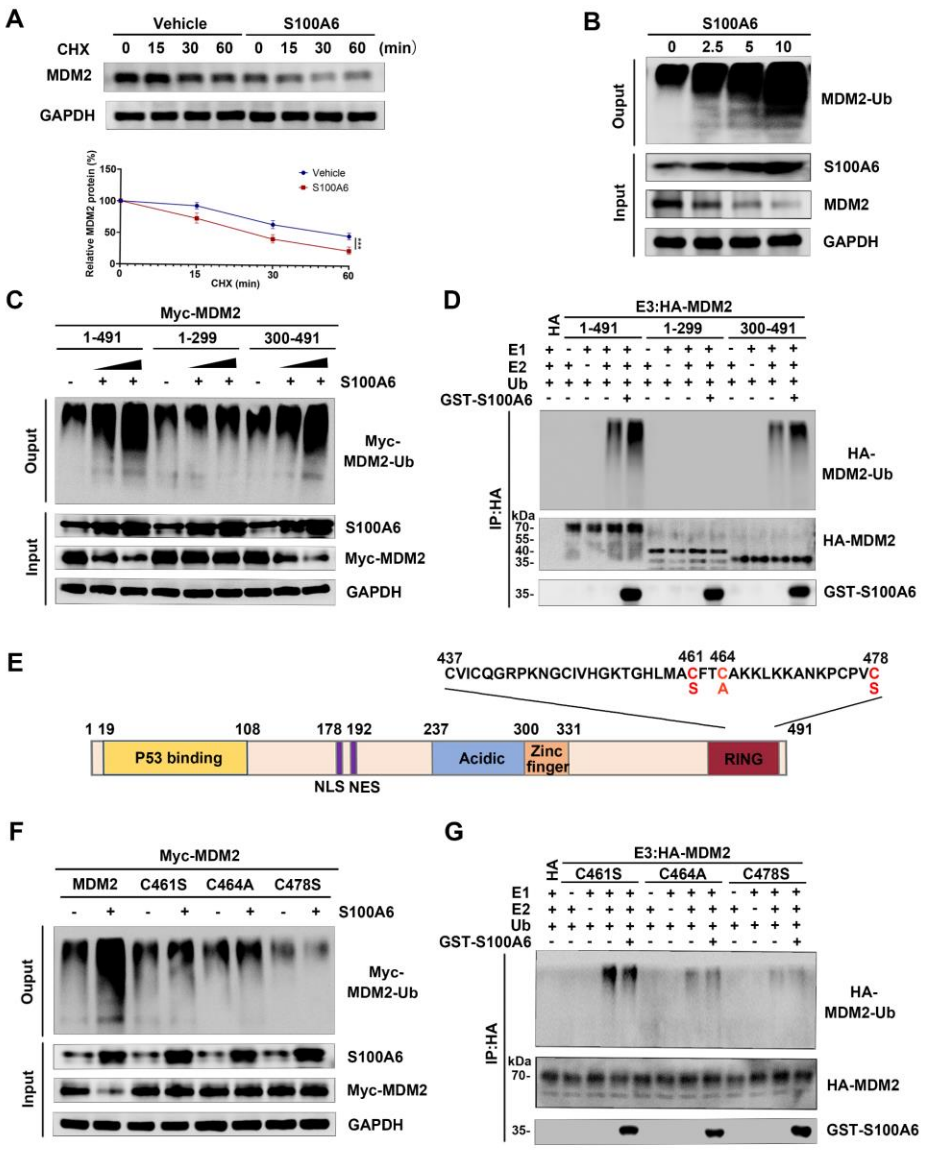
A
Vehicle
S100A6
CHX 0 15 30 60 0 15 30 60 (min)
MDM2
GAPDH
Relative MDM2 protein (%)
Vehicle
S100A6
CHX (min)
B
S100A6
0 2.5 5 10
Ouput
MDM2-Ub
Input
S100A6
MDM2
GAPDH
C
Myc-MDM2
1-491 1-299 300-491
S100A6
Ouput
Myc-MDM2-Ub
Input
S100A6
Myc-MDM2
GAPDH
D
E3:HA-MDM2
HA
1-491 1-299 300-491
E1
E2
Ub
GST-S100A6
IP:HA
HA-MDM2-Ub
kDa
HA-MDM2
GST-S100A6
E
437 461 464 478
CVICQGRPKNGCIVHGKTGHLMACFTCAKKLKKANKPCPVC
S A S
1 19 108 178 192 237 300 331 491
P53 binding
Acidic
Zinc finger
RING
NLS NES
F
Myc-MDM2
MDM2 C461S C464A C478S
S100A6
Ouput
Myc-MDM2-Ub
Input
S100A6
Myc-MDM2
GAPDH
G
E3:HA-MDM2
HA
C461S C464A C478S
E1
E2
Ub
GST-S100A6
IP:HA
HA-MDM2-Ub
kDa
HA-MDM2
GST-S100A6

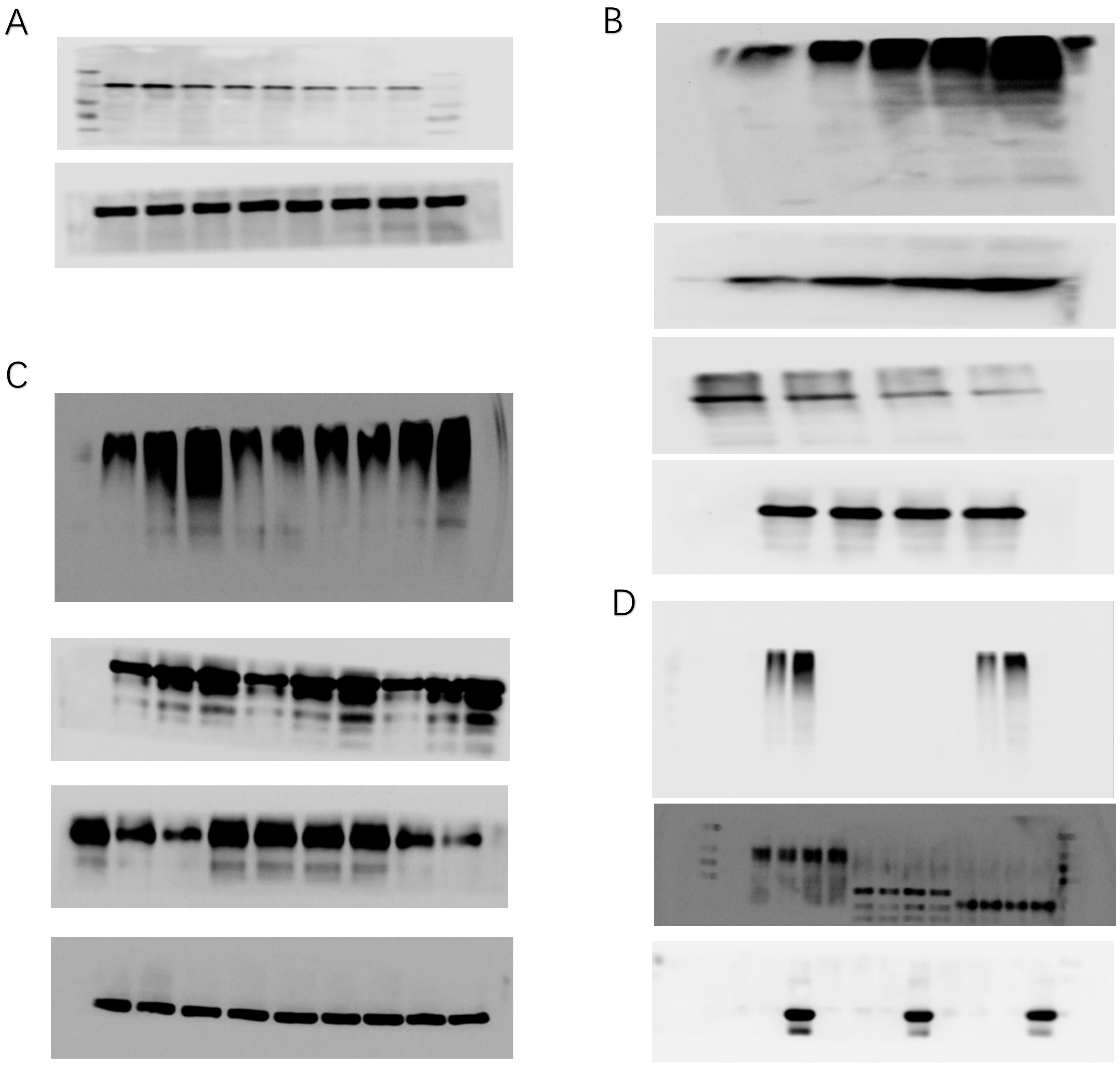
A
B
C
D

FIGURE 3

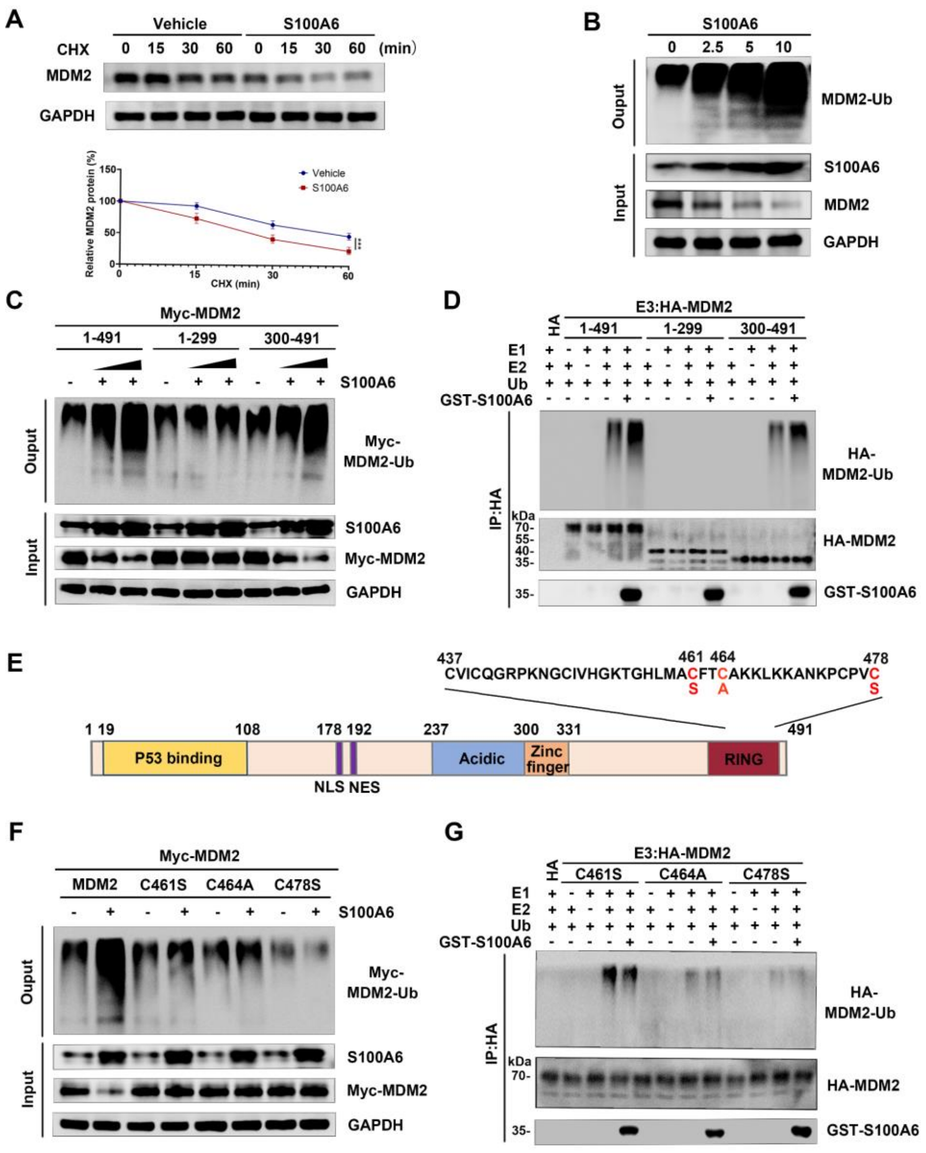
A
Vehicle
S100A6
CHX
0 15 30 60 0 15 30 60 (min)
MDM2
GAPDH
Relative MDM2 protein (%)
CHX (min)
Vehicle
S100A6
B
S100A6
0 2.5 5 10
Ouput
Input
MDM2-Ub
S100A6
MDM2
GAPDH
C
Myc-MDM2
1-491
1-299
300-491
S100A6
Ouput
Input
Myc-MDM2-Ub
S100A6
Myc-MDM2
GAPDH
D
E3:HA-MDM2
HA
1-491
1-299
300-491
E1
E2
Ub
GST-S100A6
IP:HA
kDa
HA-MDM2-Ub
HA-MDM2
GST-S100A6
E
437
CVICQGRPKNGCIVHGKTGHLMACFTCAKKLKKANKPCPVC
461 464 478
S A S
1 19 108 178 192 237 300 331 491
P53 binding
Acidic
Zinc finger
RING
NLS NES
F
Myc-MDM2
MDM2 C461S C464A C478S
S100A6
Ouput
Input
Myc-MDM2-Ub
S100A6
Myc-MDM2
GAPDH
G
E3:HA-MDM2
HA
C461S C464A C478S
E1
E2
Ub
GST-S100A6
IP:HA
kDa
HA-MDM2-Ub
HA-MDM2
GST-S100A6

F

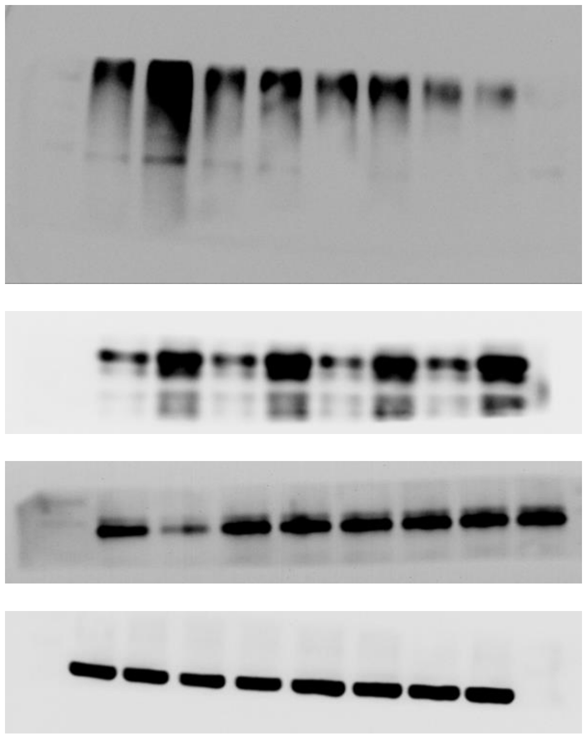

G

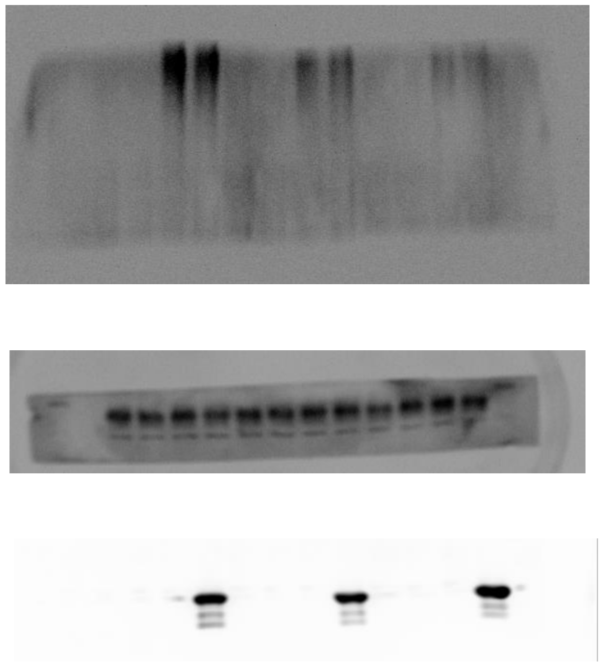

FIGURE 4

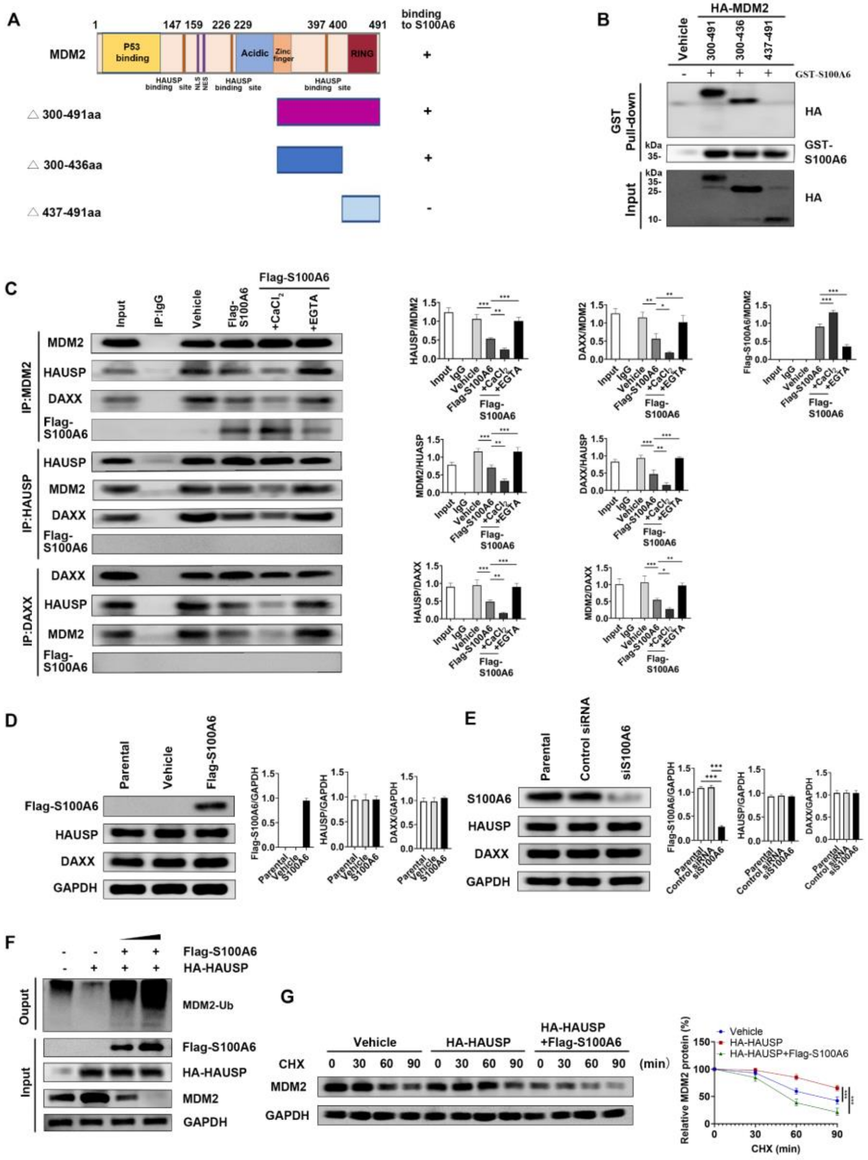
A
B
C
D
E
F
G
MDM2
HAUSP
DAXX
Flag-S100A6
GAPDH
S100A6
HA-HAUSP
MDM2-Ub
Vehicle
Parental
Control siRNA
siS100A6
HA-MDM2
GST-S100A6
GST Pull-down
Input
IP:MDM2
IP:HAUSP
IP:DAXX
CHX
HA-HAUSP+Flag-S100A6

B

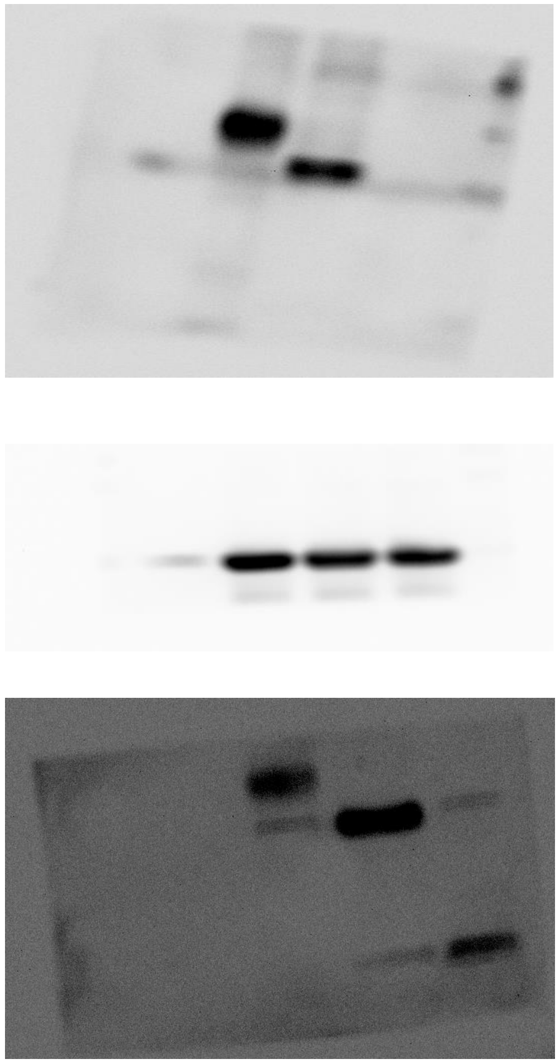

D

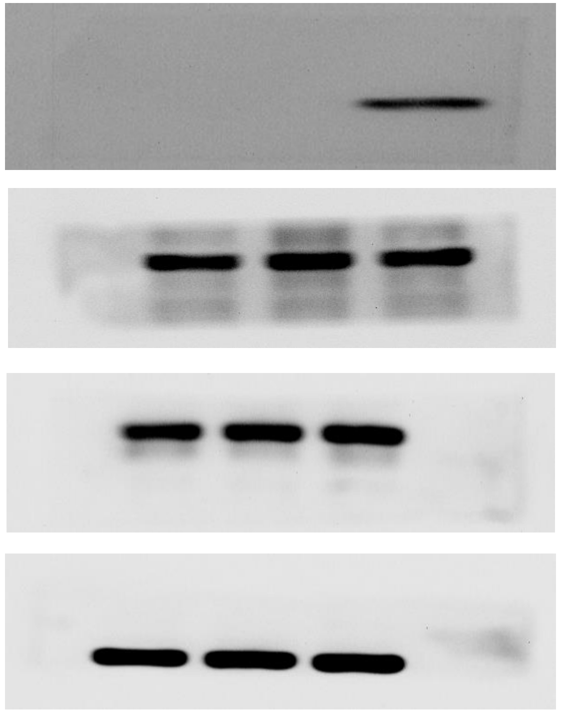

E

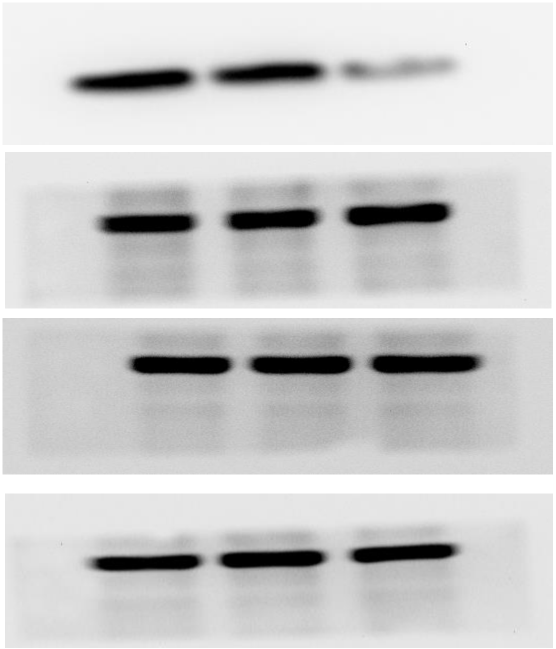

FIGURE 4

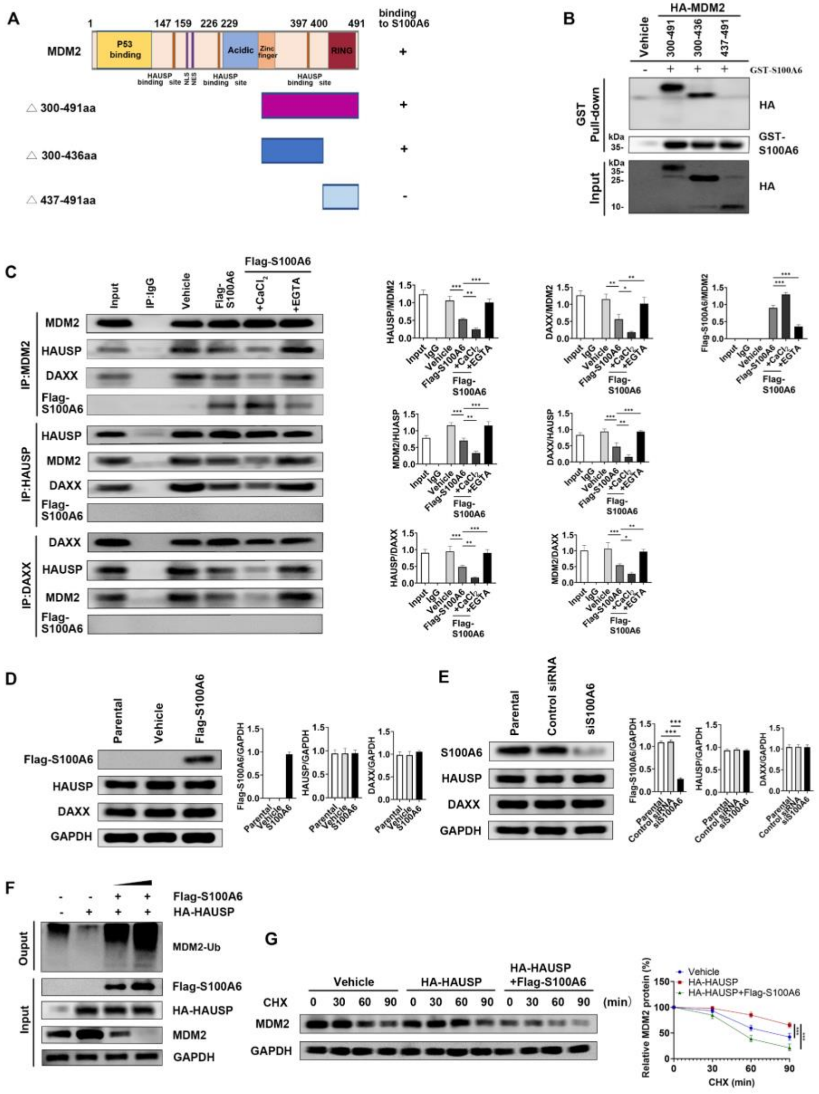

C

IP:MDM2

HAUSP

DAXX

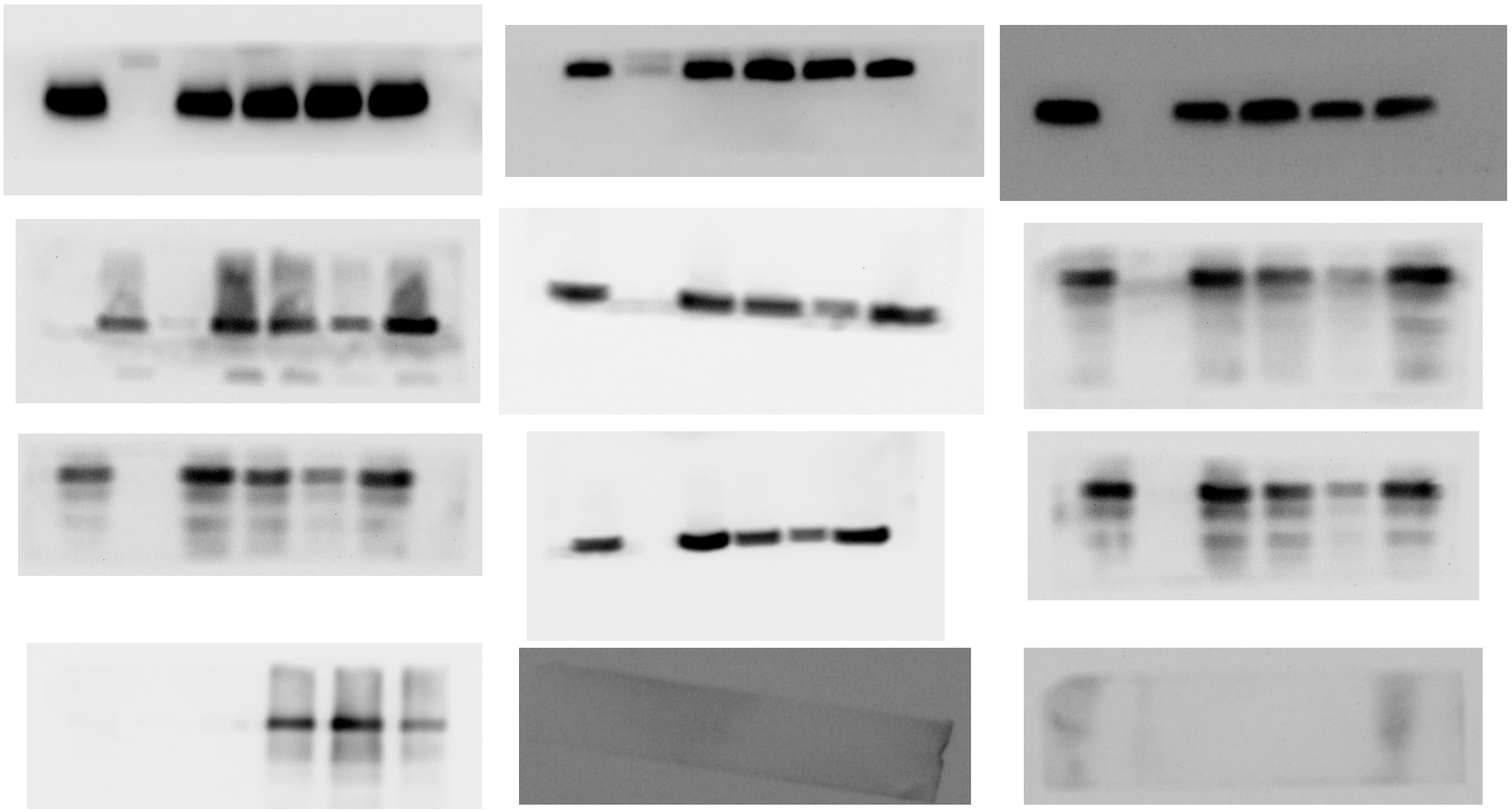

FIGURE 4

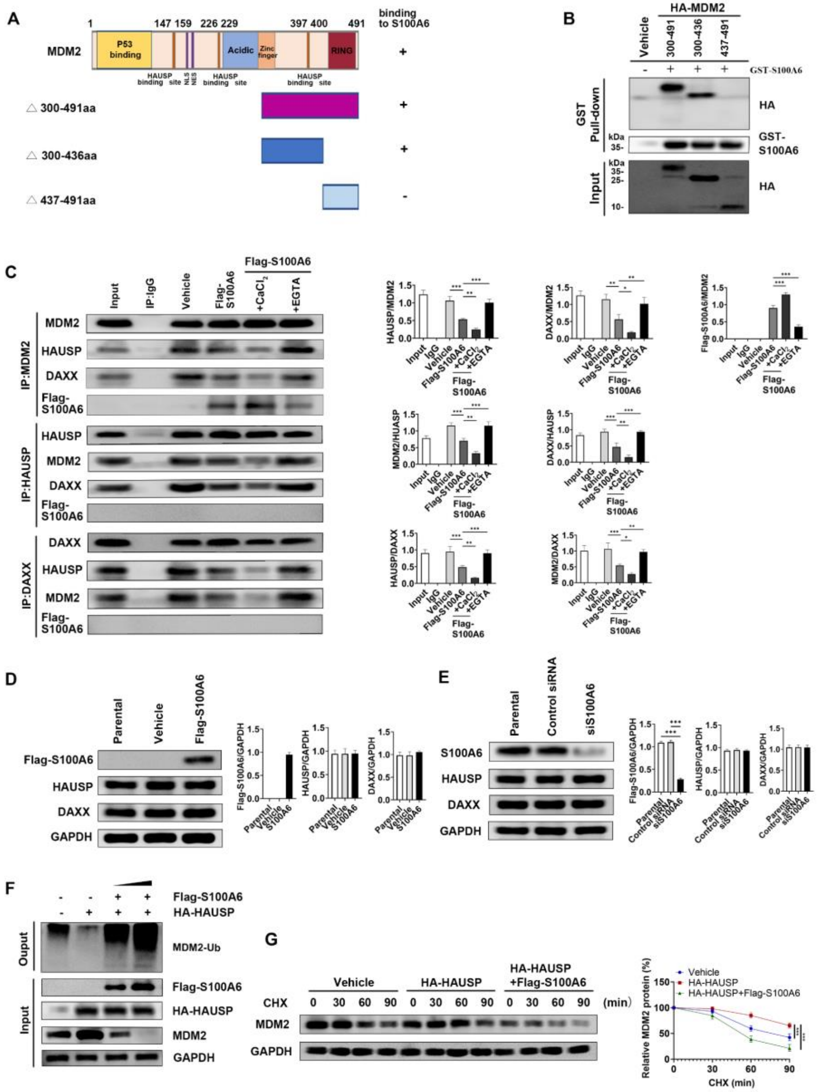
A
MDM2
binding to S100A6
P53 binding
Acidic
Zinc finger
RING
HAUSP binding site
△ 300-491aa
△ 300-436aa
△ 437-491aa
B
HA-MDM2
Vehicle
300-491
300-436
437-491
GST-S100A6
GST Pull-down
Input
HA
C
Flag-S100A6
Input
IP:IgG
Vehicle
+CaCl2
+EGTA
MDM2
HAUSP
DAXX
IP:MDM2
IP:HAUSP
IP:DAXX
D
Parental
Flag-S100A6
GAPDH
E
Control siRNA
siS100A6
S100A6
F
HA-HAUSP
MDM2-Ub
Ouput
G
HA-HAUSP
+Flag-S100A6
CHX
(min)
Relative MDM2 protein (%)
CHX (min)

F

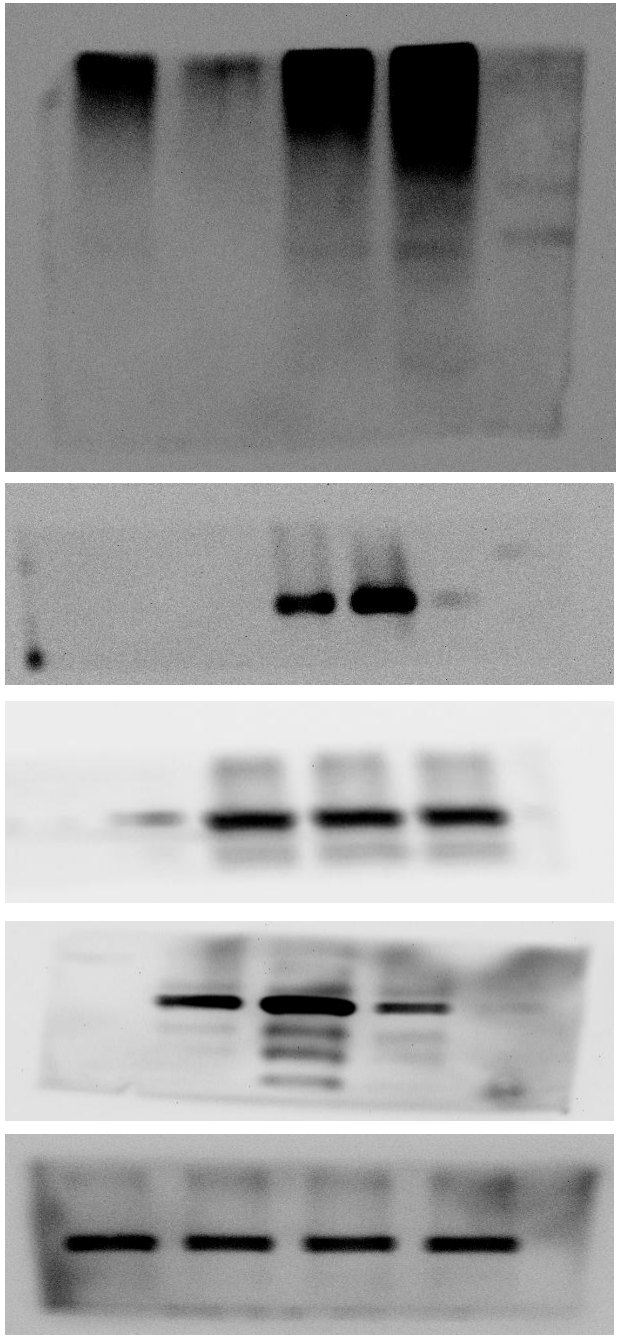

G

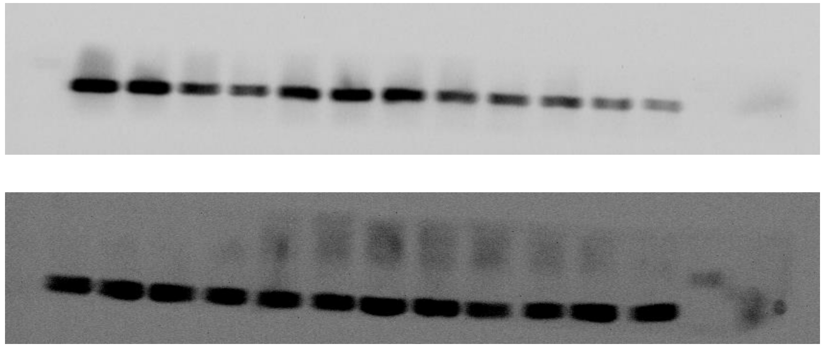

FIGURE 5

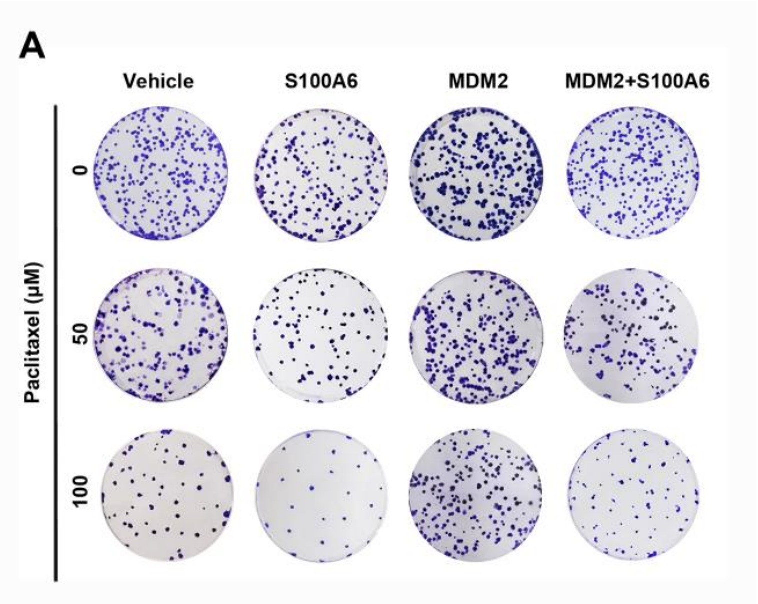

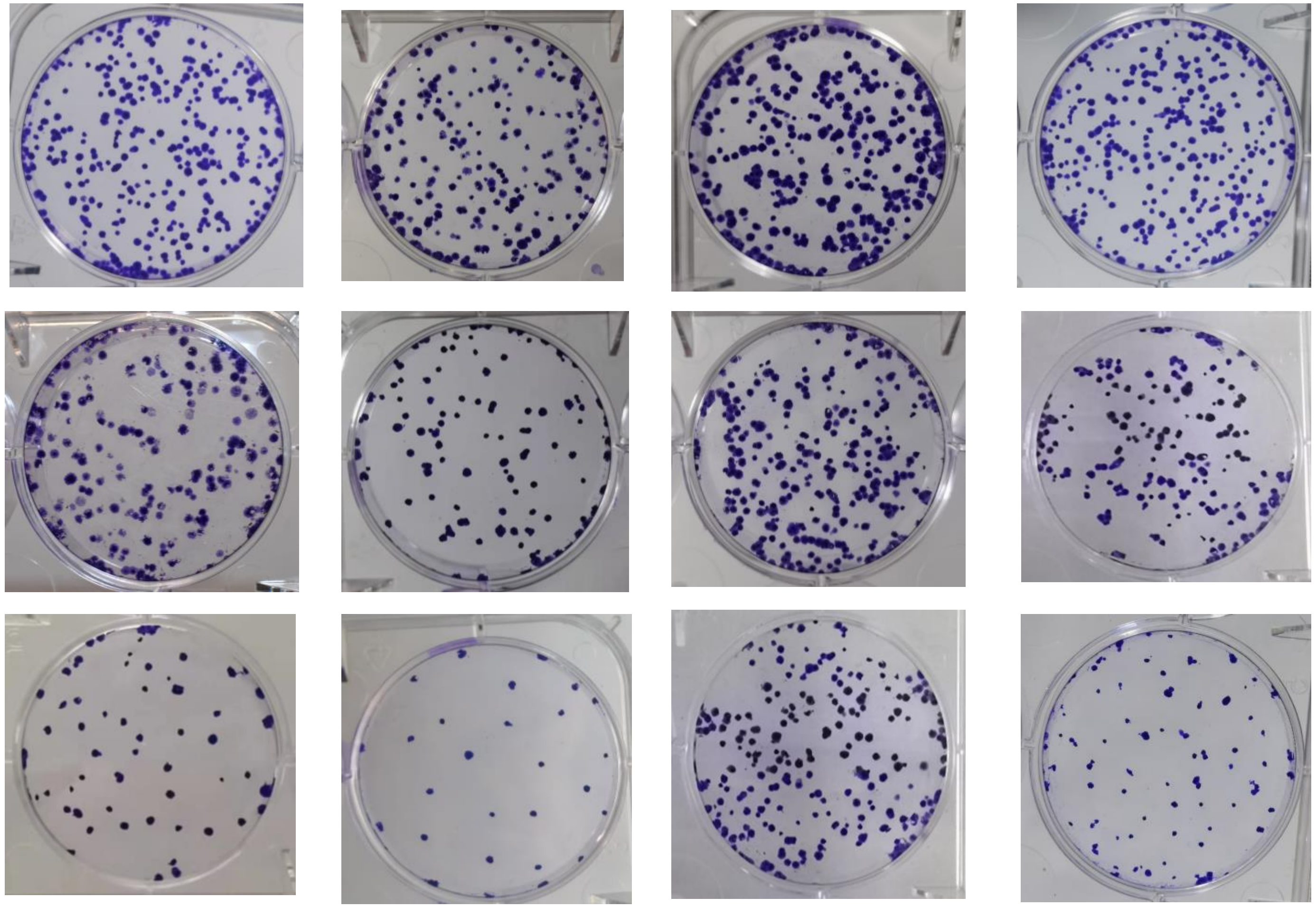

FIGURE 6

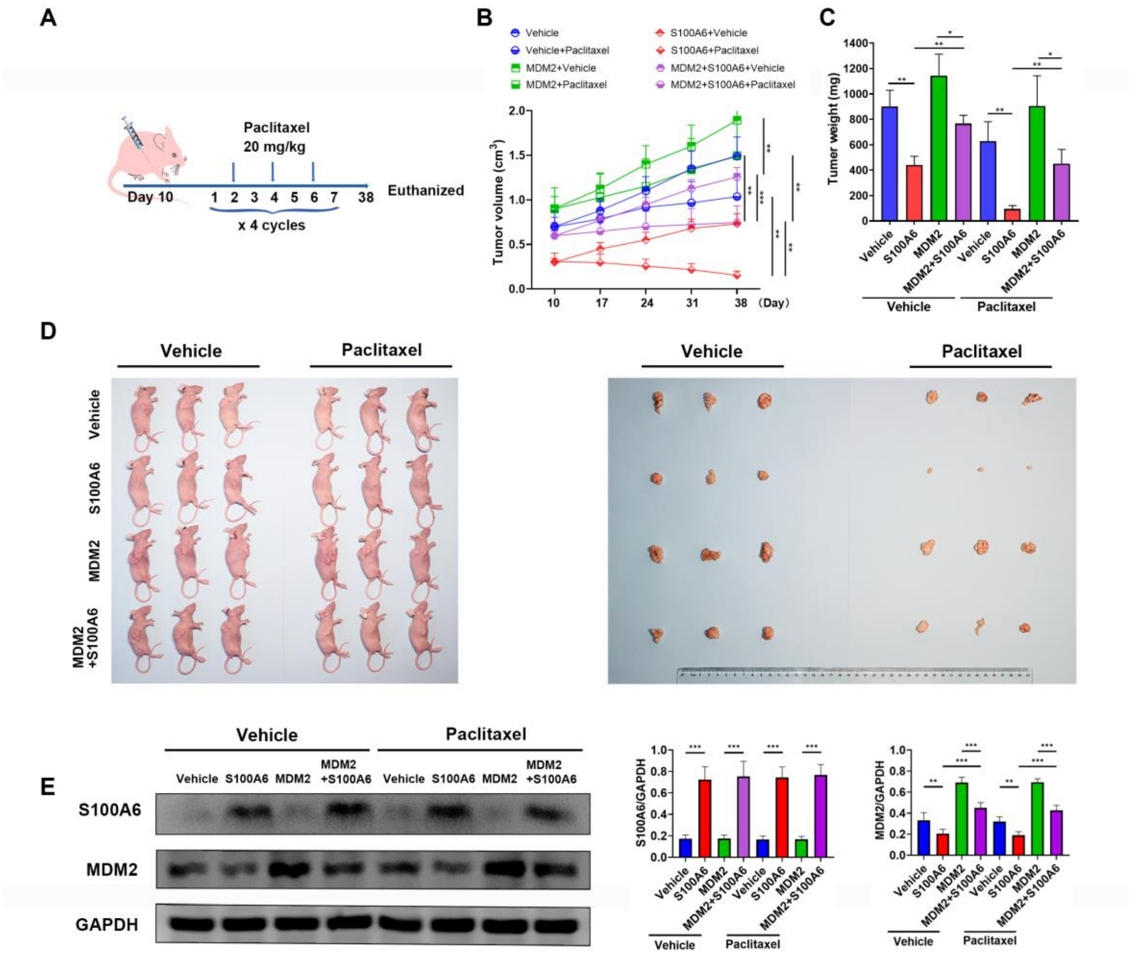
A
Paclitaxel
20 mg/kg
Day 10
1 2 3 4 5 6 7 38
Euthanized
x 4 cycles
B
Vehicle
Vehicle+Paclitaxel
MDM2+Vehicle
MDM2+Paclitaxel
S100A6+Vehicle
S100A6+Paclitaxel
MDM2+S100A6+Vehicle
MDM2+S100A6+Paclitaxel
Tumor volume (cm3)
(Day)
C
Tumer weight (mg)
Vehicle
S100A6
MDM2
MDM2+S100A6
Paclitaxel
D
Vehicle
Paclitaxel
Vehicle
S100A6
MDM2
MDM2 +S100A6
E
Vehicle
Paclitaxel
Vehicle S100A6 MDM2 MDM2 +S100A6 Vehicle S100A6 MDM2 MDM2 +S100A6
S100A6
MDM2
GAPDH
S100A6/GAPDH
MDM2/GAPDH

E

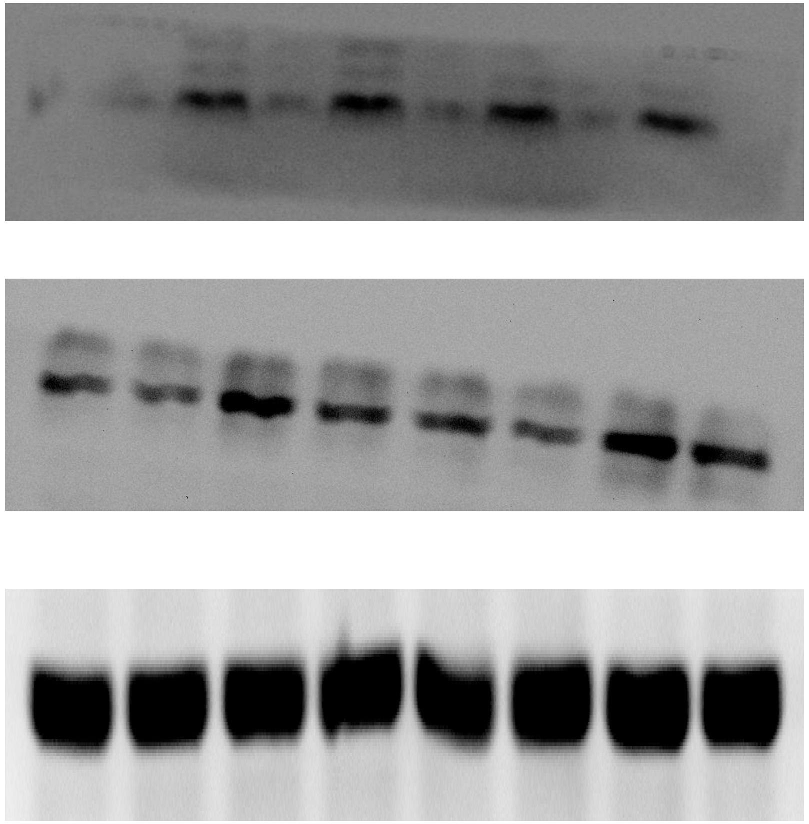

Supplement: Supplementary file 1 — Additional file 1. Raw data for western blot and colony formation assay. [file 13058_2023_1657_MOESM1_ESM.pdf]
